# Transcription factor *c-Maf* deletion improves streptozotocin-induced diabetic nephropathy by directly regulating *Sglt2* and *Glut2*

**DOI:** 10.1172/jci.insight.163306

**Published:** 2023-03-22

**Authors:** Mitsunori Fujino, Naoki Morito, Takuto Hayashi, Masami Ojima, Shun Ishibashi, Akihiro Kuno, Seizo Koshiba, Kunihiro Yamagata, Satoru Takahashi

**Affiliations:** 1 Department of Anatomy and Embryology, Faculty of Medicine;; 2 PhD Program in Human Biology, School of Integrative and Global Majors;; 3 Department of Nephrology, Faculty of Medicine; and; 4 Doctoral Program in Biomedical Sciences, Graduate School of Comprehensive Human Sciences, University of Tsukuba, Ibaraki, Japan.; 5 Tohoku Medical Megabank Organization and; 6 Advanced Research Center for Innovations in Next-Generation Medicine (INGEM), Tohoku University, Sendai, Japan.; 7 Laboratory Animal Resource Center,; 8 Life Science Center for Survival Dynamics, Tsukuba Advanced Research Alliance (TARA),; 9 International Institute for Integrative Sleep Medicine (WPI-IIIS), and; 10 Transborder Medical Research Center, Faculty of Medicine, University of Tsukuba, Ibaraki, Japan.

**Keywords:** Nephrology, Chronic kidney disease, Diabetes

## Abstract

The transcription factor c-Maf has been widely studied and has been reported to play a critical role in embryonic kidney development; however, the postnatal functions of c-Maf in adult kidneys remain unknown as *c-Maf*–null C57BL/6J mice exhibit embryonic lethality. In this study, we investigated the role of c-Maf in adult mouse kidneys by comparing the phenotypes of tamoxifen-inducible (TAM-inducible) *c-Maf*–knockout mice (*c-Maf^fl/fl^*; *CAG-Cre-ER*^TM^ mice named “*c-Maf*^ΔTAM^”) with those of *c-Maf^fl/fl^* control mice, 10 days after TAM injection [TAM(10d)]. In addition, we examined the effects of *c-Maf* deletion on diabetic conditions by injecting the mice with streptozotocin, 4 weeks before TAM injection. *c-Maf*^ΔTAM^ mice displayed primary glycosuria caused by sodium-glucose cotransporter 2 (*Sglt2*) and glucose transporter 2 (*Glut2*) downregulation in the kidneys without diabetes, as well as morphological changes and life-threatening injuries in the kidneys on TAM(10d). Under diabetic conditions, *c-Maf* deletion promoted recovery from hyperglycemia and suppressed albuminuria and diabetic nephropathy by causing similar effects as did *Sglt2* knockout and SGLT2 inhibitors. In addition to demonstrating the potentially unique gene regulation of c-Maf, these findings highlight the renoprotective effects of *c-Maf* deficiency under diabetic conditions and suggest that *c-Maf* could be a novel therapeutic target gene for treating diabetic nephropathy.

## Graphical abstract

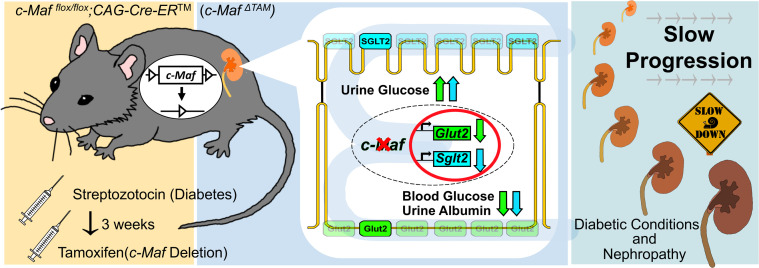

## Introduction

c-Maf is a member of the large Maf family of basic leucine zipper transcription factors. The leucine zipper domain mediates dimer formation, and its basic region binds to the Maf recognition element (MARE) in genomic DNA. There are 3 types of MAREs, namely TRE-type, CRE-type, and half-MARE (1). In addition to these domains, large Maf family members are characterized by the presence of an N-terminal acidic transactivation domain (2). The functions of c-Maf have been widely studied, and it has been reported to play critical roles in the embryonic stage, including in the differentiation of T lymphocyte subsets (3, 4), differentiation involving γ-crystallin synthesis in lens fiber cells (5, 6), differentiation of hypertrophic chondrocytes during endochondral bone development (7, 8), and development of the embryonic kidney and liver cells. In mice, *c-Maf* is first expressed on embryonic day 16 in the proximal tubules (9); however, the postnatal functions of c-Maf in adult kidneys remain unclear as *c-Maf*–null mice in a C57BL/6J background exhibit embryonic lethality (10). Therefore, to investigate the functions of c-Maf in adult kidneys, we previously generated tamoxifen-inducible (TAM-inducible) *c-Maf*–knockout (*c-Maf^fl/fl^*; *CAG-Cre-ER*^TM^) mice, which we named “*c-Maf*^ΔTAM^” mice (11).

Transporters mediate the translocation of substrates across biological membranes and are widely expressed throughout the body, including the liver, brain, and kidney. There are 2 main transporter superfamilies, namely the ATP-binding cassette (ABC) superfamily and the solute carrier (SLC) superfamily. ABC transporters use energy from ATP hydrolysis and function as efflux transporters, whereas SLC transporters are associated with the cellular uptake of small molecules. Transporters from both families have attracted considerable attention in drug development as over 80 SLC transporters are known to be involved in monogenic disorders (12) and, thus, have therapeutic potential.

The kidneys reabsorb glucose through specific transporters, including sodium-glucose cotransporters (SGLTs) and glucose transporters (GLUTs) (13). SGLT2 is considered a major glucose reabsorption transporter as the reabsorption ratio of SGLT2-filtered glucose is 80%–90% in the proximal tubules (14, 15). Importantly, the pharmacological inhibition of SGLT2 reduces hyperglycemia and glomerular hyperfiltration by suppressing renal glucose reabsorption to increase the excretion of urinary glucose, thereby reducing the risk of progression to chronic kidney disease (CKD) (16–19). SGLT2 inhibitors have, therefore, received increasing attention as a target for a new class of blood glucose–lowering drugs that have been approved to treat type 2 diabetes mellitus and CKD.

In addition, it was recently reported that induction of renal *Glut2* deficiency reversed hyperglycemia in streptozotocin-induced (STZ-induced) mouse models (20). Glut2 is localized in renal proximal tubule cells and normally affects the basolateral efflux of the reabsorbed glucose from the tubular cells back into the circulation. Hyperglycemia induces Glut2 expression in the kidney, and its increased expression contributes to the development of diabetic kidney disease (DKD) in mice. Renal proximal tubule cell–specific ablation of *Glut2* has been demonstrated to ameliorate the progress of DKD in mice because of suppression of cannabinoid-1 receptor stimulation and mTOR complex 1 (mTORC1) activity (21, 22). Therefore, in addition to *Sglt2*, *Glut2* can be established as a target gene for the treatment of diabetes. However, the physiological contributions of Sglt2 and Glut2 in the kidney to systemic glucose homeostasis have been not completely established; therefore, it is important to elucidate the mechanisms underlying the function of these transporters.

This study was aimed at investigating the role of c-Maf in adult mice by exploring the specific phenotypes of *c-Maf*^ΔTAM^ mice and characterizing the associated c-Maf transcriptional profiles using RNA sequencing (RNA-Seq) and quantitative polymerase chain reaction (qPCR). In addition, we evaluated the direct binding of c-Maf with *Sglt2* and *Glut2* and explored the effects of *c-Maf* deletion on diabetes and kidney injury. Overall, in this study, we found potentially novel aspects of *c-Maf* regulation in the kidneys and provided causal insights into c-Maf regulation and the phenotypes of *c-Maf*^ΔTAM^ mice, including urinary features and improvements in diabetic conditions and nephropathy progression.

## Results

### c-Maf^ΔTAM^ mice develop severe renal glycosuria, basic aminoaciduria, and albuminuria.

**Figure 1:**
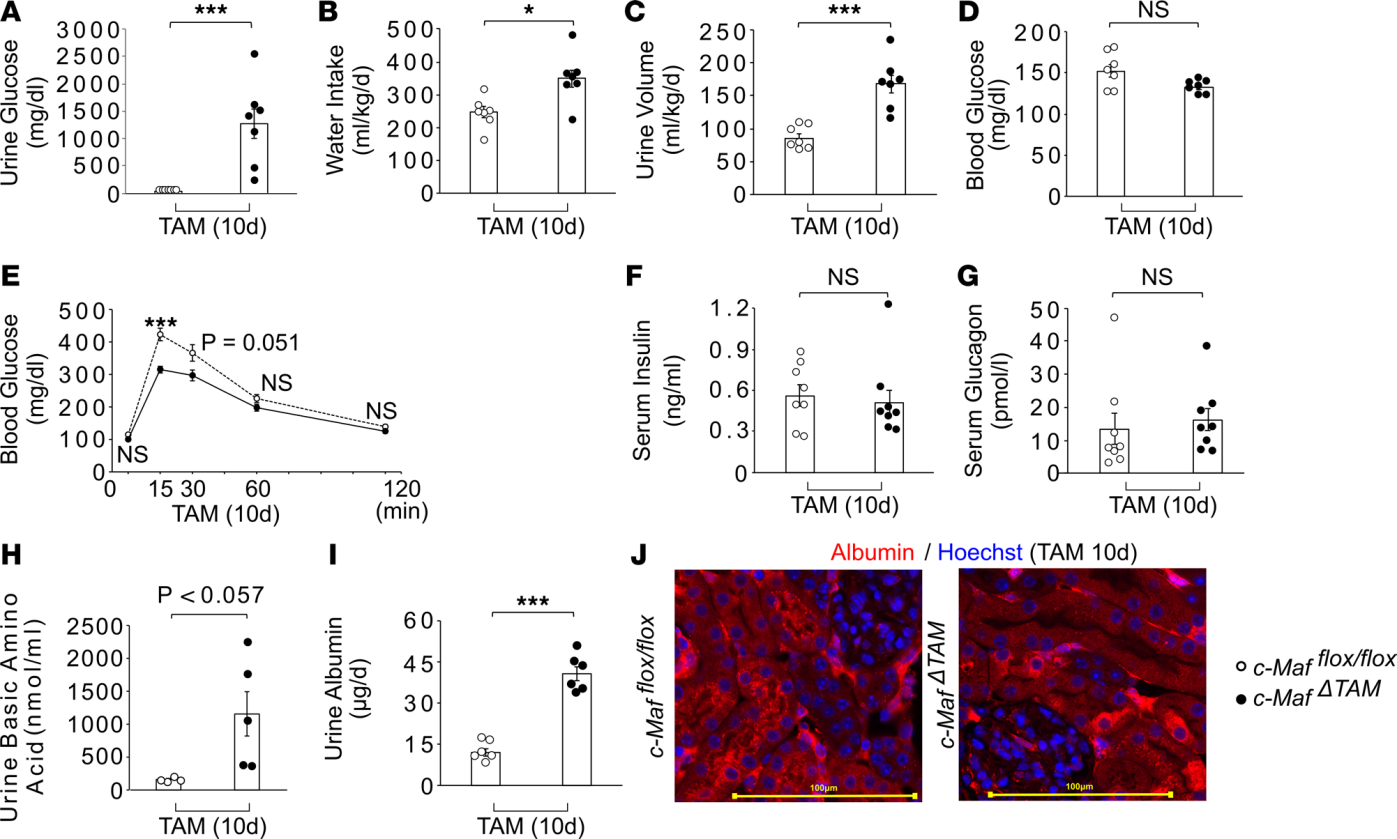
*c-Maf^ΔTAM^* mice develop severe renal glycosuria, basic aminoaciduria, and albuminuria. Changes in (**A**) urine glucose, (**B**) water intake, and (**C**) urine volume in *c-Maf^ΔTAM^* and *c-Maf^fl/fl^* groups (*n* = 7 per group). *c-Maf^ΔTAM^* group showed higher urinary glucose level, water intake, and urine volume. Loss of *c-Maf* did not alter glucose homeostasis, as determined by (**D**) 4-hour fasting blood glucose level, (**E**) glucose tolerance test after 10 hours of fasting, (**F**) serum insulin level, and (**G**) serum glucagon level. Glucose tolerance was examined before and up to 120 minutes after glucose solution injection (*n* = 11 in *c-Maf^fl/fl^* and *n* = 15 in *c-Maf^ΔTAM^* groups). (**H**) Urinary basic amino acid levels increased in the *c-Maf^ΔTAM^* mice compared with the *c-Maf^fl/fl^* mice. (**I**) *c-Maf^ΔTAM^* group showed higher urinary albumin levels than the *c-Maf^fl/fl^* group (*n* = 6 per group). (**J**) IHC staining for albumin expression in the *c-Maf^ΔTAM^* group showed decreased granular signals in the proximal tubules compared with the *c-Maf^fl/fl^* group (*n* = 3 per group). **A** analyses were performed on TAM(10d). Scale bars: 100 μm. **A**–**I** were presented as the mean and the standard error of the mean (SEM). To assess whether differences between *c-Maf^ΔTAM^* and *c-Maf^fl/fl^* mice were statistically significant, a minimum of 3 biological replicates were analyzed using Welch’s *t* test, and a *P* value < 0.05 was considered significant. *P < 0.05, and ***P < 0.001. White circles: *c-Maf^fl/fl^* groups, and black circles: *c-Maf^ΔTAM^* groups.

To explore the roles of c-Maf in adults, we analyzed the urine and blood components of 8-week-old *c-Maf*^ΔTAM^ and *c-Maf^fl/fl^* (control) mice, 10 days after TAM injection [TAM(10d)]. First, we found that urinary glucose levels were sharply increased in *c-Maf*^ΔTAM^ mice compared with those in *c-Maf^fl/fl^* mice on TAM(10d) (Figure 1A). In addition, water intake and urine volume were higher in *c-Maf*^ΔTAM^ mice than in the control mice (Figure 1, B and C). Together, these 3 conditions are typical signs of diabetes; however, blood glucose levels in *c-Maf*^ΔTAM^ mice were only slightly lower than in the control mice (Figure 1D). In addition, intraperitoneal glucose tolerance tests (IPGTTs), serum insulin and glucagon levels, and insulin receptor substrate 1 (*IRS1*) and *IRS2* mRNA expression levels were comparable between the 2 groups (Figure 1, E–G, and Supplemental Figure 1, A and B; supplemental material available online with this article; https://doi.org/10.1172/jci.insight.163306DS1). Moreover, to examine c-Maf expression, morphological changes upon *c-Maf* deletion, and c-Maf function in the pancreas, IHC staining, hematoxylin and eosin (HE) staining, and analysis of serum amylase, whose value changes when there is an abnormality in the pancreas, were performed. The IHC staining revealed c-Maf expression around the exocrine cells, such as the macrophages and interstitial area, but not in the islet cells (Supplemental Figure 1C). Although its expression in *c-Maf*^ΔTAM^ mice decreased compared with that in *c-Maf^fl/fl^* mice, there were no morphological changes and no significant difference in amylase levels in the serum under the feeding conditions compared with those in *c-Maf^fl/fl^* mice (Supplemental Figure 1, D and E).

In addition, we examined the liver and small intestine, along with the pancreas, because the roles of c-Maf in adult liver and small intestine were recently reported (23, 24). In the case of liver, c-Maf was expressed around the hepatic cells, such as sinusoidal cells and macrophages (Supplemental Figure 1F). Although there was a tendency for decreased c-Maf expression, no morphological changes were observed (Supplemental Figure 1G). In addition, there were no significant differences in serum aspartate transaminase and alanine transaminase levels, which are criteria for liver function (Supplemental Figure 1, H and I). In fact, time-specific *c-Maf* loss did not alter the fractal dimension patterning and did not induce liver damage, such as fibrosis, under basal conditions (23). Considering our data and the results reported previously, we concluded that *c-Maf* deletion does not have primary and critical effects on the function and morphology of the liver in this study.

In the case of small intestine, there was a tendency for decreased c-Maf expression (Supplemental Figure 1J); however, *c-Maf*^ΔTAM^ mice did not show morphological changes in the small intestine compared with that in *c-Maf^fl/fl^* mice (Supplemental Figure 1K). In fact, although in enterocytes, the expression of c-Maf plays an important role in nutrient absorption, TAM-induced *c-Maf* deletion in the entire intestinal epithelium did show comparable change in the body weight and unaltered nutrient absorption capacity with no compensatory increase in other MAF family members (24). Considering our data and the result reported previously, we concluded that *c-Maf* deletion does not have primary and critical effects on the function and morphology of the small intestine as well as of the pancreas and liver in this study. Taken together, we concluded that the significant increase in urinary glucose levels was due to primary glycosuria, not diabetes.

In addition to primary glycosuria, the *c-Maf*^ΔTAM^ mice showed a tendency for increased urinary basic amino acid levels compared with *c-Maf^fl/fl^* mice on TAM(10d) (Figure 1H); however, the total serum concentrations of these amino acids did not differ significantly (Supplemental Figure 1L). High urinary basic amino acid levels are a typical feature of Fanconi renotubular syndrome (FRTS; Online Mendelian Inheritance in Man [OMIM]: 134600, 613388, 615605, 616026, 618913) (25). Consistently, the mice displayed some other characteristics of FRTS, including increased renal sodium and a tendency for increased urinary phosphate levels, potassium, and calcium wasting; a tendency for lower urinary uric acid levels; and no significant differences in serum sodium and potassium levels (Supplemental Figure 1, M–S), indicating that FRTS was incomplete in *c-Maf*^ΔTAM^ mice.

We also found that urinary albumin levels were higher on TAM(10d) in *c-Maf*^ΔTAM^ mice than in *c-Maf^fl/fl^* mice because of reduced albumin reabsorption in the proximal tubules, as indicated by immunohistochemistry (IHC) staining (Figure 1, I and J). Since high urinary albumin levels are a typical marker of nephropathy, we performed histological and biochemical analysis on the kidneys. Physiological serum creatinine and urea nitrogen (UN) levels did not differ significantly between the *Maf*^ΔTAM^ and *c-Maf^fl/fl^* mice (Supplemental Figure 1, T and U). Furthermore, histological analysis using HE and periodic acid–Schiff staining as well as electron microscopy images revealed no significant structural changes in the glomeruli or brush border membranes of the proximal tubules in *c-Maf*^ΔTAM^ and *c-Maf^fl/fl^* mice on TAM(10d) (Supplemental Figure 1V).

Given that *c-Maf*^ΔTAM^ mice can survive for at least 2 years following TAM injection (*n* = 6, data not shown), it is possible that *c-Maf* loss leads to renal glycosuria, basic aminoaciduria and albuminuria without diabetes, complete FRTS, morphological changes, and life-threatening kidney injuries.

### Relationship between c-Maf and renal features in c-Maf^ΔTAM^ mice.

**Figure 2:**
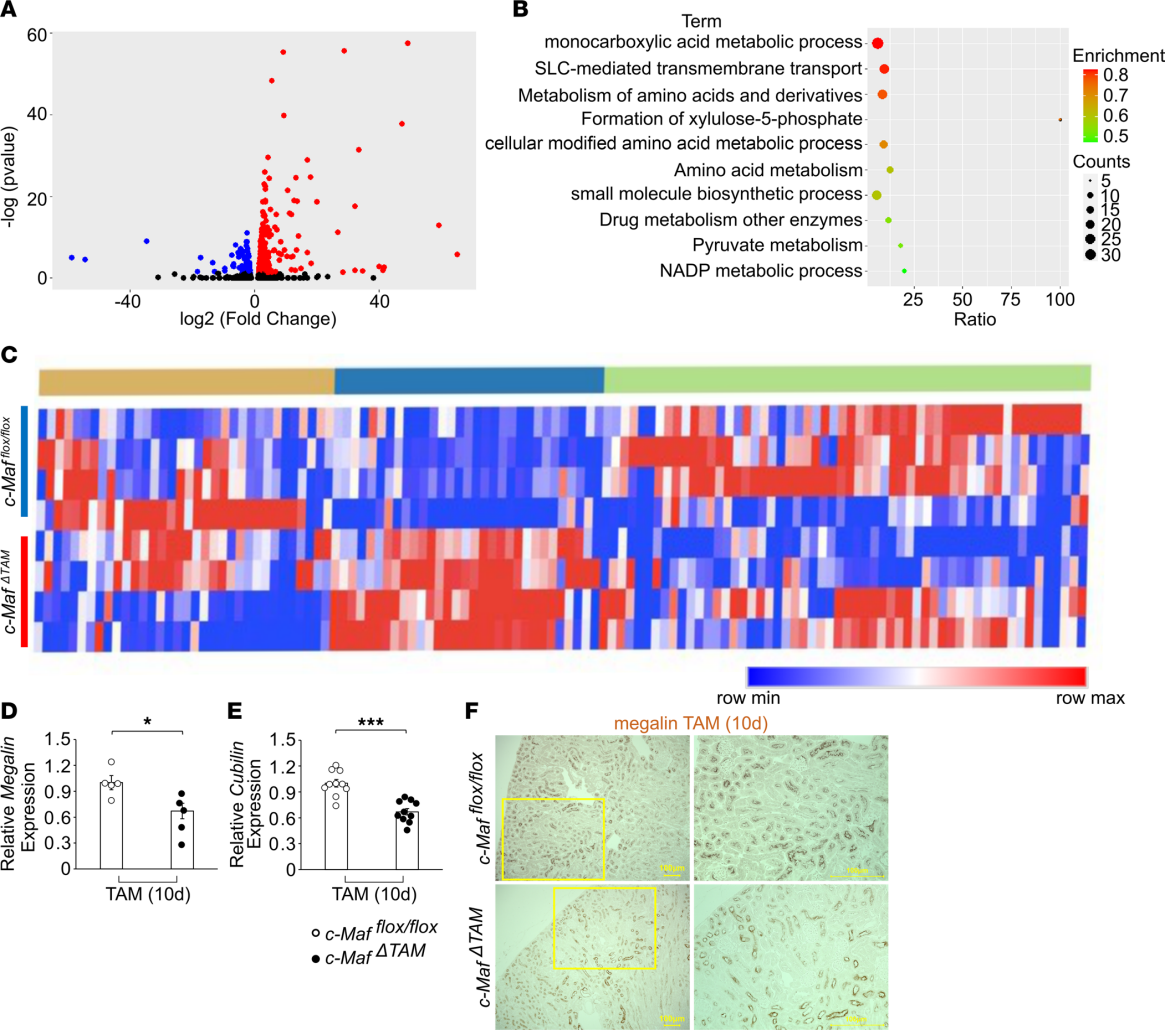
RNA-Seq analysis of the relationship between c-Maf and renal features. (**A**) Volcano plot of overall gene expression: 261 genes were upregulated (red) and 113 genes were downregulated (blue) in *c-Maf^ΔTAM^* mice. (**B**) Top 10 pathways of the DEGs in *c-Maf^ΔTAM^* mice. (**C**) Heatmap of the expression of 123 genes related to SLC-mediated transmembrane transport between *c-Maf^ΔTAM^* and *c-Maf^fl/fl^* mice (*n* = 4 per group). (**D**) *Megalin* and (**E**) *cubilin* gene expression levels were lower in *c-Maf^ΔTAM^* mice than in *c-Maf^fl/fl^* mice (*n* = 10 per group). (**F**) A tendency for decreased megalin protein expression levels in *c-Maf^ΔTAM^* mice than in *c-Maf^fl/fl^* mice (*n* = 4 per group). Scale bars: 100 μm. For RNA-Seq transcriptome analysis, gene expression values were calculated using the quantile method. Gene expression was compared between *c-Maf^fl/fl^* control mice and *c-Maf^ΔTAM^* mice using the Empirical Analysis DGE tool (edgeR test) in the CLC Genomics Workbench (Version 10.1.1; QIAGEN). False discovery rate (FDR) adjustments (81) were applied to correct for multiple testing, and DEGs were identified using an FDR cutoff of 0.05. **D** and **E** were presented as the mean and the standard error of the mean (SEM). To assess whether differences between *c-Maf^ΔTAM^* and *c-Maf^fl/fl^* mice were statistically significant, a minimum of 3 biological replicates were analyzed using Welch’s *t* test, and a *P* value < 0.05 was considered significant. *P < 0.05, and ***P < 0.001. White circles: *c-Maf^fl/fl^* groups, and black circles: *c-Maf^ΔTAM^* groups.

To determine why glycosuria, aminoaciduria, and albuminuria occurred in *c-Maf*^ΔTAM^ mice from the perspective of genetic variation, we performed RNA-Seq and qPCR analyses in both the groups. A total of 374 differentially expressed genes (DEGs) were detected between *c-Maf*^ΔTAM^ and *c-Maf^fl/fl^* mice (Figure 2A), among which 261 were upregulated and 113 were downregulated. Pathway analysis of these DEGs indicated a strong relationship between *c-Maf* and SLC-mediated transmembrane transport (Figure 2B). To obtain further insights into the regulation of *c-Maf* in specific clusters, we constructed a k-means clustering heatmap (Figure 2C) and selected 1 cluster with clear differences in both groups (Supplemental Figure 2A). This cluster included 2 glucose transporters, Slc5a2 (Sglt2) and Slc2a2 (Glut2), through which renally filtered glucose is reabsorbed in the proximal tubules (13, 26), suggesting that primary glycosuria in *c-Maf*^ΔTAM^ mice is caused by decreased expression of these transporters on TAM(10d). In addition, the cluster included several amino acid transporters that were downregulated in *c-Maf*^ΔTAM^ mice, which may result in aminoaciduria (Supplemental Figure 2B). In terms of albuminuria, qPCR analysis suggested that the gene expression of megalin (*Lrp2*) and cubilin (*Cubn*), which are responsible for albumin reabsorption in proximal tubules, was downregulated in *c-Maf*^ΔTAM^ mice (Figure 2, D and E). In addition, IHC staining revealed a tendency for decreased megalin expression in proximal tubules of *c-Maf*^ΔTAM^ mice compared with that in *c-Maf^fl/fl^* mice; no negative feedback was observed at least at the mRNA level (Figure 2F). Indeed, previous studies using *Lrp2*- and *Cubn*-deficient mice have reported no significant morphological changes in the kidneys due to deficiency of these 2 genes (27, 28). Together, these results elucidate *c-Maf* gene regulation in kidney proximal tubules and demonstrate that *c-Maf* deletion leads to renal glycosuria, basic aminoaciduria, and albuminuria by downregulating the gene expression of several transporters in the kidney.

### c-Maf is a potentially novel transcription factor that regulates Sglt2 and Glut2.

**Figure 3:**
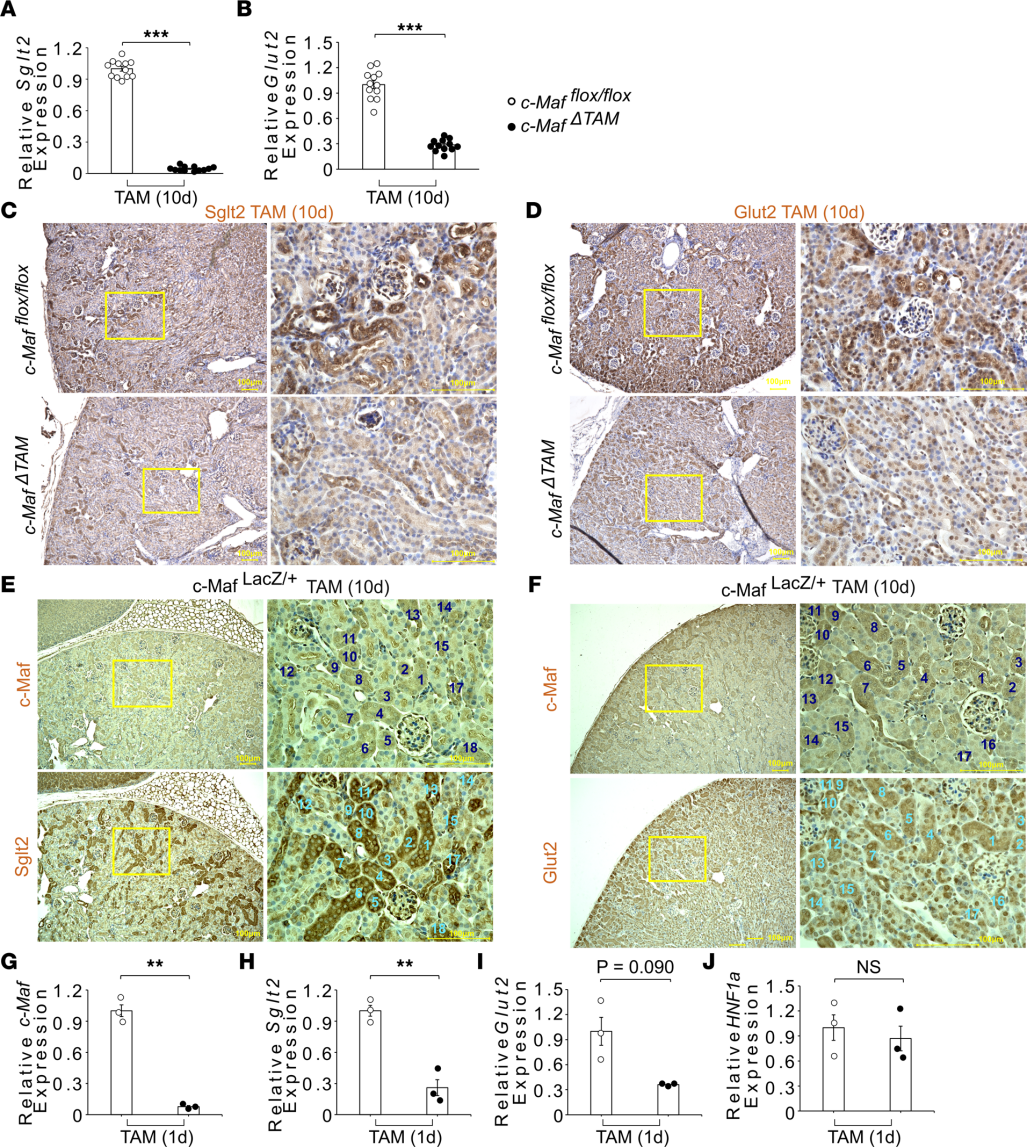
*c-Maf ^ΔTAM^* mice show decreased Sglt2 and Glut2 gene and protein expression. (**A**) *Sglt2* and (**B**) *Glut2* mRNA levels were analyzed in kidney tissues from *c-Maf^fl/fl^* and *c-Maf^ΔTAM^* mice using qPCR (*n* = 12 per group) on TAM(10d). (**C**) Sglt2 and (**D**) Glut2 protein expression was decreased in the kidney cortex of *c-Maf^ΔTAM^* mice compared with *c-Maf^fl/fl^* mice (*n* = 4 per group). (**E** and **F**) c-Maf colocalization with Sglt2 and Glut2 in consecutive sections (*n* = 3). (**G**) *c-Maf*, (**H**) *Sglt2*, (**I**) *Glut2*, and (**J**) *HNF1α* mRNA level was analyzed in kidney tissues from *c-Maf^fl/fl^* and *c-Maf^ΔTAM^* mice using qPCR (*n* = 3 per group) at 1 day after the second TAM injection. Blue numbers indicate the colocalization of c-Maf with the transporters Sglt2 and Glut2. For IHC analyses, 3-week-old *c-Maf^ΔTAM^* and *c-Maf^fl/fl^* mice were injected with TAM to verify Sglt2 and Glut2 protein expression. The colocalization of the transporters with c-Maf was verified using 6-week-old *c-Maf^LacZ/+^* mice (*n* = 3 per group). **A**, **B**, and **G**–**J** were presented as the mean and the standard error of the mean (SEM). To assess whether differences between *c-Maf^ΔTAM^* and *c-Maf^fl/fl^* mice were statistically significant, a minimum of 3 biological replicates were analyzed using Welch’s *t* test, and a *P* value < 0.05 was considered significant. Scale bars: 100 μm. **P < 0.01, and ***P < 0.001. White circles: *c-Maf^fl/fl^* groups, and black circles: *c-Maf^ΔTAM^* groups.

To verify the direct regulation of *Sglt2* and *Glut2* by *c-Maf*, we evaluated the RNA expression levels of these transporters using qPCR. The expression of *Sglt2* and *Glut2* was significantly lower in the kidneys of *c-Maf*^ΔTAM^ mice, consistent with the observed reduction in *c-Maf* expression (Figure 3, A and B, and Supplemental Figure 3A). Moreover, *Glut1* expression was unchanged and *Sglt1* expression was slightly increased, likely to compensate for *Sglt2* downregulation (Supplemental Figure 3, B and C) (29). IHC analysis further verified that the expression of Sglt2 and Glut2 proteins was substantially decreased in *c-Maf*^ΔTAM^ mice compared with that in the *c-Maf^fl/fl^* control mice (Figure 3, C and D). Moreover, localization of the 2 transporters was consistent with that of c-Maf in the proximal tubules of the kidneys in *c-Maf^LacZ/+^* mice (Figure 3, E and F). Next, we examined *c-Maf*, *Sglt2*, *Glut2*, and hepatocyte nuclear factor 1α (*HNF1α*) in the kidneys at 1 day after the second TAM injection [TAM(1d)]. *c-Maf* and *Sglt2* expression in *c-Maf*^ΔTAM^ mice was significantly reduced, and there was tendency for decreased *Glut2* expression even 1 day after the second injection compared with that in the *c-Maf^fl/fl^* mice (Figure 3, G–I); in contrast, there was no significant difference in *HNF1α* expression in both groups on TAM(10d) (Figure 3J). In fact, in the previous study, it was concluded that renal *Glut2* deficiency reduced the expression of Sglt2 on day 14 after the third TAM injection via downregulation of the transcription factor HNF1α (20). However, in this study, *c-Maf* deletion significantly decreased the expression of *Sglt2* on only a day after the second TAM injection, suggesting that the time lag in the reduction of *Sglt2* expression between previous studies and this study indicates direct regulation of *Sglt2* by c-Maf. Therefore, these results suggest that c-Maf directly controls the expression of Sglt2 and Glut2 in the kidney.

### c-Maf directly controls the expression of Sglt2 and Glut2.

**Figure 4:**
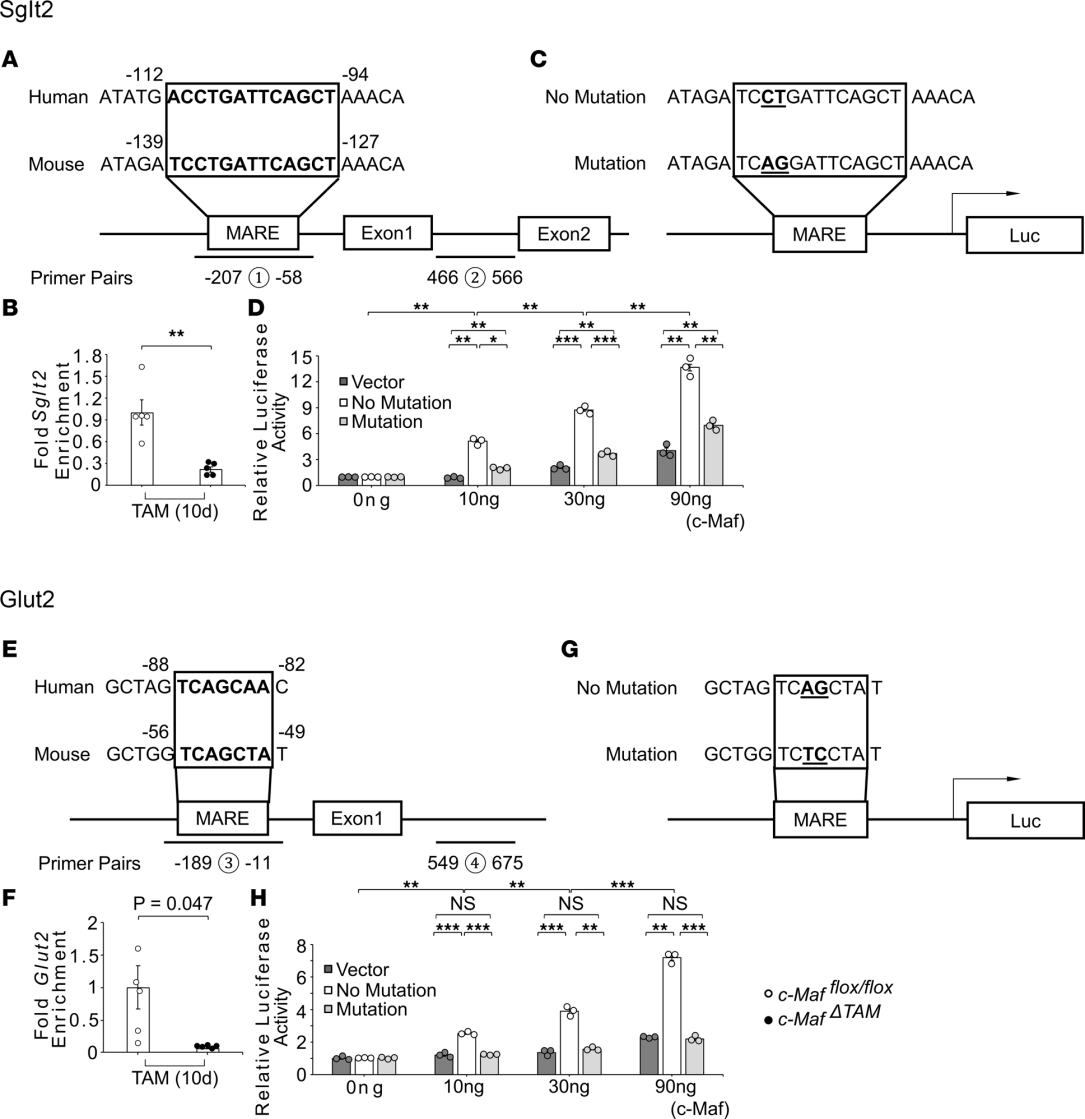
c-Maf as a potentially novel *Sglt2*- and *Glut2*-regulating transcription factor. ChIP assay and luciferase reporter assay revealed the direct regulation of *Sglt2* and *Glut2* by c-Maf. Quantitative ChIP confirmed direct binding of c-Maf to MARE sites in (**A** and **B**) *Sglt2* and (**E** and **F**) *Glut2* loci; ChIP assay was performed upstream of the transcription start sites (primer pairs 1 for *Sglt2* and 3 for *Glut2*), and intron sites as an additional negative control (primer pairs 2 for *Sglt2* and 4 for *Glut2*). The luciferase reporter system examined the activation of (**C** and **D**) *Sglt2* and (**G** and **H**) *Glut2*. Dual-luciferase reporter plasmids with or without a mutation in *Sglt2* and *Glut2* were cotransfected with the vectors carrying *c-Maf* (0–90 ng). Experiments were repeated 3 times. **B** and **F** were presented as the mean and the standard error of the mean (SEM). To assess whether differences between *c-Maf^ΔTAM^* and *c-Maf^fl/fl^* mice were statistically significant, a minimum of 3 biological replicates were analyzed using Welch’s *t* test, and a *P* value < 0.05 was considered significant. Holm-Šídák corrections were applied for multiple statistical tests in **D** and **H**. *P < 0.05, **P < 0.01, and ***P < 0.001. White circles: *c-Maf^fl/fl^* groups, and black circles: *c-Maf^ΔTAM^* groups.

Since the sequences upstream of the *Sglt2* and *Glut2* transcription start sites contain a TRE-type MARE and half-MARE, respectively, we performed chromatin immunoprecipitation (ChIP) and luciferase assays to verify that c-Maf controls *Sglt2* and *Glut2* by directly binding to their MARE sites (Figure 4, A, C, E, and G). The primer pairs constructed for these assays, including those for MAREs, are shown in Supplemental Tables 1 and 2. The ChIP assays revealed that the gene regulatory region amplification signals for *Sglt2* and *Glut2* were significantly reduced in the kidneys of *c-Maf*^ΔTAM^ mice on TAM(10d) compared with that in *c-Maf^fl/fl^* mice, suggesting that c-Maf bound to the MAREs in the *Sglt2* and *Glut2* gene regulatory regions (Figure 4, B and F). Luciferase assays confirmed that c-Maf significantly increased the activities of *Sglt2* and *Glut2* in the absence of MARE mutations but that their activities showed significantly lower responsiveness toward c-Maf in the presence of MARE mutations (Figure 4, D and H). Therefore, these results verify that c-Maf directly regulates *Sglt2* and *Glut2* and is associated with their expression in adult mouse kidneys, resulting in severe glycosuria.

### Improvements in diabetic conditions and kidney function in c-Maf^ΔTAM^ mice.

The clinical hallmarks of diabetes mellitus and diabetic nephropathy with CKD progression include hyperglycemia, a high glomerular filtration rate, and increased urinary albumin excretion (30–34). It has been reported that STZ-treated mice exhibit hyperglycemia, hyperfiltration, and high urinary albumin levels compared with untreated mice and that these aspects are improved in *Sglt2*-knockout mice (35) and by SGLT2 inhibitors (36–38). Therefore, we examined whether *c-Maf* deletion had similar effects on diabetes, blood glucose levels, creatinine clearance rate (Ccr), osmolar clearance, serum creatinine and UN levels, and urinary albumin levels in STZ-induced diabetic *c-Maf*^ΔTAM^ mice. Details of the experimental protocol are provided in Supplemental Figure 4A. Although we were unable to distinguish differences in c-Maf localization in the proximal tubules, *c-Maf* gene expression was significantly lower in the kidneys of *c-Maf^fl/fl^* mice after STZ(7w) TAM(3w) than after TAM(8w) without STZ (Supplemental Figure 4B), and c-Maf protein expression was significantly decreased in the kidneys of *c-Maf*^ΔTAM^ mice after STZ(12w) TAM(8w) compared with that in *c-Maf^fl/fl^* mice (Supplemental Figure 4, C and D), likely due to proximal tubular damage. In addition, *Sglt2* and *Glut2* gene expression was significantly decreased in the kidneys of *c-Maf^fl/fl^* mice after STZ(7w) TAM(3w) versus after TAM(8w) without STZ (Supplemental Figure 4, E and F), probably due to the decreasing *c-Maf* level. While there were no significant differences in body weight on STZ(4w) TAM(0w) between the groups, we observed a tendency for increased body weight in *c-Maf*^ΔTAM^ mice on STZ(12w) TAM(8w) compared with that in the control mice, with no significant difference in food intake (Supplemental Figure 4, G and H).

**Figure 5:**
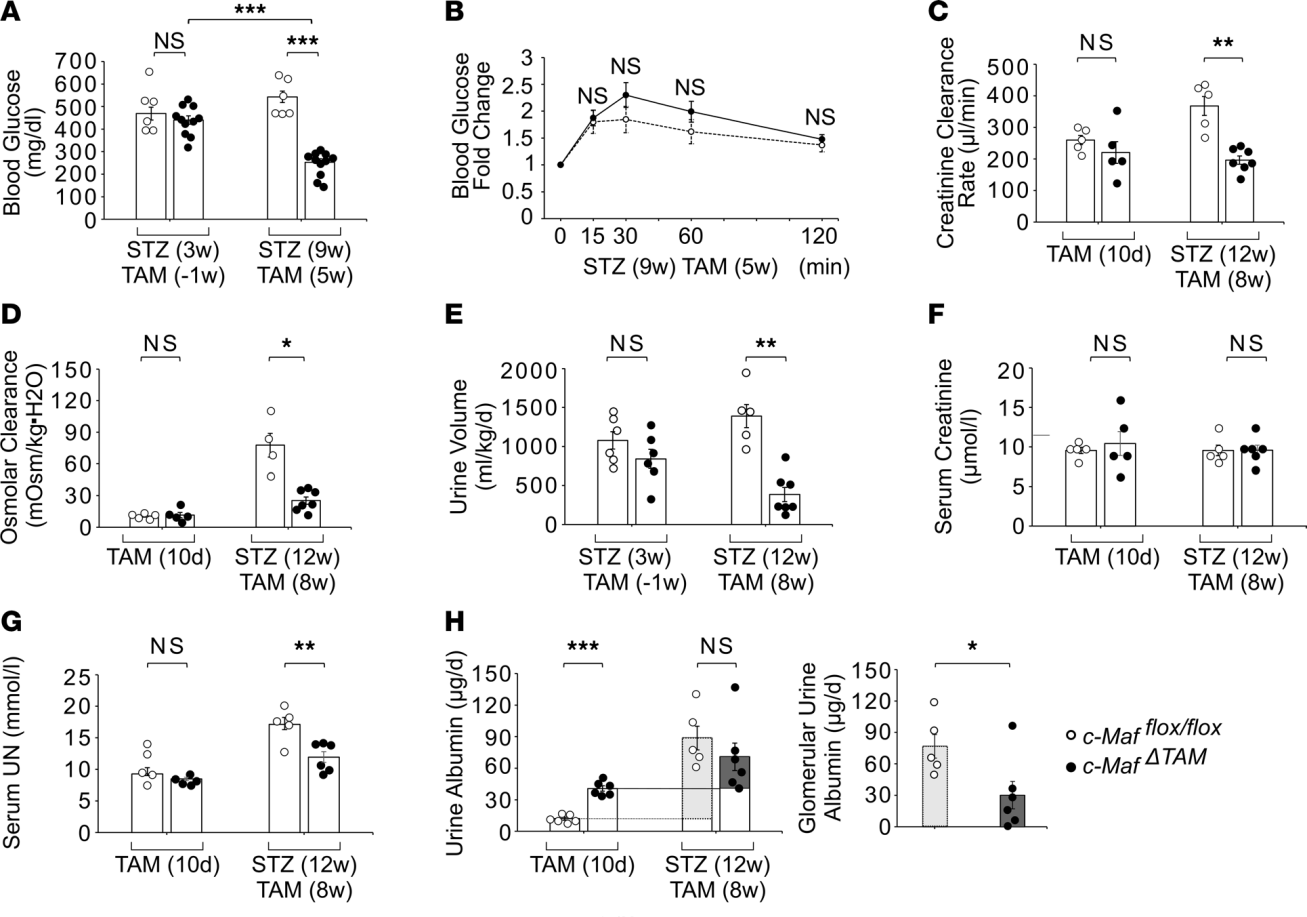
*c-Maf* loss attenuates diabetic conditions. (**A**) *c-Maf^ΔTAM^* mice (*n* = 11) displayed significantly lower blood glucose levels after 10-hour fasting than *c-Maf^fl/fl^* mice (**B**) but no significant difference in glucose intolerance between 2 groups. *n* = 6 on STZ(9w) TAM(5w). (**C**) Creatinine clearance rate, (**D**) osmolar clearance, and (**E**) urine volume were reduced in *c-Maf^ΔTAM^* mice, as a result of improved glomerular hyperfiltration, compared with control mice. [**C**: *n* = 5 per group on TAM(10d); *c-Maf^fl/fl^*, *n* = 5 and *c-Maf^ΔTAM^*, *n* = 7 on STZ(12w). **D**: *n* = 5 per group on TAM(10d); *c-Maf^fl/fl^*, *n* = 4 and *c-Maf^ΔTAM^*, *n* = 7 on STZ(12w). **E**: *n* = 6 per group on STZ(3w) TAM(-1w); *c-Maf^fl/fl^*, *n* = 5 and *c-Maf^ΔTAM^*, *n* = 7 on STZ(12w) TAM(8w)]. Different mice were assessed on TAM(10d) and STZ(12w) TAM(8w). c-Maf deletion attenuated diabetic kidney nephropathy, as indicated by an improvement in (**F**) serum creatinine and (**G**) serum UN levels in *c-Maf^ΔTAM^* mice compared with *c-Maf^fl/fl^* mice, as well as (**H**) urinary albumin levels on TAM(10d) (left) and STZ(12w) TAM(8w) (center) and glomerular urinary albumin levels on STZ(12w) TAM(8w) (right). [**F** and **G**: *n* = 5 per group on TAM(10d); *c-Maf^fl/fl^*, *n* = 5 and *c-Maf^ΔTAM^*, *n* = 6 on STZ(12w). **H**: *n* = 8 per group on TAM(10d); *c-Maf^fl/fl^*, *n* = 5 and *c-Maf^ΔTAM^*, *n* = 6 on STZ(12w)]. **B**–**H** were presented as the mean and the standard error of the mean (SEM). To assess whether differences between *c-Maf^ΔTAM^* and *c-Maf^fl/fl^* mice were statistically significant, a minimum of 3 biological replicates were analyzed using Welch’s *t* test, and a *P* value < 0.05 was considered significant. Holm-Šídák corrections were applied for multiple statistical tests in **A**. *P < 0.05, **P < 0.01, ***P < 0.001. White circles: *c-Maf^fl/fl^* groups, and black circles: *c-Maf^ΔTAM^* groups.

As shown in Figure 5A, blood glucose levels in *c-Maf*^ΔTAM^ mice were significantly reduced, 9 weeks after STZ and 5 weeks after TAM injection [STZ(9w) TAM(5w)], compared with those in *c-Maf*^ΔTAM^ mice, 3 weeks after STZ and 1 week before TAM injection [STZ(3w) TAM(–1w)], despite the gradual progression of hyperglycemia in *c-Maf^fl/fl^* mice. In fact, *c-Maf*^ΔTAM^ mice showed higher urinary glucose levels than the control mice on STZ(12w) TAM(8w) (Supplemental Figure 4I). Although glucose intolerance was not attenuated in *c-Maf*^ΔTAM^ mice on STZ(9w) TAM(5w) because of STZ-induced diabetes, similar to that in *c-Maf^fl/fl^* mice (Figure 5B), Ccr, osmolar clearance, and blood pressure were decreased in *c-Maf*^ΔTAM^ mice on STZ(12w) TAM(8w), suggesting that the suppression of glomerular hyperfiltration may decrease Ccr, osmolar clearance, and blood pressure, resulting in decreased urine volume (Figure 5, C–E, and Supplemental Figure 4J) (35). While serum creatinine levels did not change between *c-Maf*^ΔTAM^ and *c-Maf^fl/fl^* mice, serum UN levels improved (Figure 5, F and G).

Next, we examined glomerular urinary albumin levels, which indicate the total amount of albumin leaked from the glomerulus and are a typical marker of diabetic nephropathy. No significant differences were observed in urinary albumin levels, which represent the total amount of nephron albumin on STZ(12w) TAM(8w) between the 2 groups (Figure 5H); however, urinary albumin levels were higher in *c-Maf*^ΔTAM^ mice on TAM(10d) than in *c-Maf^fl/fl^* mice because of the downregulation of *Lrp2* and *Cubn* genes, resulting in reduced albumin reabsorption in proximal tubules (Figure 5H and Figure 2D). Using a previously reported method (39) and the difference between total albumin levels on STZ(12w) TAM(8w) and urinary albumin levels on TAM(10d) (Figure 5H), we found that glomerular urine albumin levels were significantly reduced in *c-Maf*^ΔTAM^ mice on STZ(12w) TAM(8w) compared with those in *c-Maf^fl/fl^* mice (Figure 5H). This decrease in glomerular urinary albumin levels in *c-Maf*^ΔTAM^ mice on STZ(12w) TAM(8w) could indicate improvements in diabetic nephropathy. In addition, these results demonstrated similar attenuations in hyperglycemia and hyperfiltration compared with those in *Sglt2*-knockout mice and in those treated with SGLT2 inhibitors and STZ injection, as well as lowered glomerular albuminuria in *c-Maf*^ΔTAM^ mice. Therefore, these findings suggest that c-Maf loss exerts similar effects on hyperglycemia, other diabetic conditions, and kidney nephropathy as Sglt2 knockout and SGLT2 inhibitors.

### Improvement of diabetic nephropathy in c-Maf^ΔTAM^ mice.

**Figure 6:**
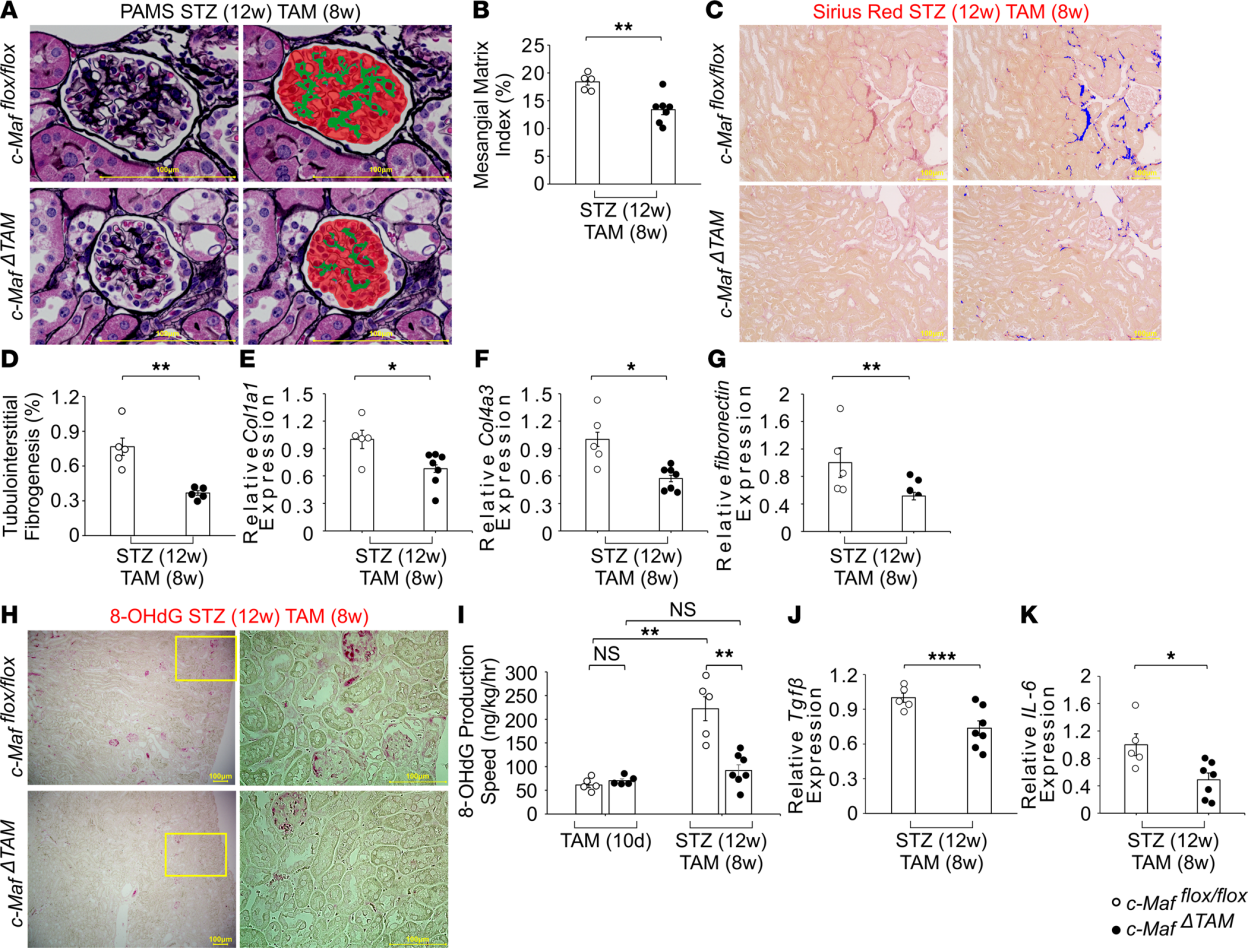
Diabetic kidney injury improves in *c-Maf^ΔTAM^* mice through reduced oxidative stress and inflammatory cytokine levels. Decreased (**A** and **B**) PAMS-positive mesangial matrix index and (**C** and **D**) Sirius red–positive area indicate the suppression of diabetic kidney injury in *c-Maf^ΔTAM^* mice (**A** and **B**: *c-Maf^fl/fl^*, *n* = 5; *c-Maf^ΔTAM^*, *n* = 7. **C** and **D**: *n* = 5 per group). Kidney sections collected on STZ(12w) TAM(8w) were used for both staining procedures. The increased ratio of mesangial matrix and fibrogenesis in *c-Maf^ΔTAM^* mice was ameliorated compared with *c-Maf^fl/fl^* mice. The mesangial matrix index represents the ratio of the mesangial matrix area (green) to the tuft area (glomerulus in red). Tubulointerstitial fibrogenesis lesions, defined as Sirius red–positive areas (blue), were measured to detect fibrogenesis and expressed as a percentage of the total area. (**E**) *Col1a1*, (**F**) *Col4a3*, and (**G**) *fibronectin* expression on STZ(12w) TAM(8w) (*c-Maf^fl/fl^*, *n* = 5; *c-Maf^ΔTAM^*, *n* = 7). (**H**) IHC staining of 8-OHdG distribution in the kidneys STZ(12w) TAM(8w) (*n* = 4 per group). (**I**) Urinary 8-OHdG levels indicate reduced oxidative stress in the kidneys of *c-Maf^ΔTAM^* mice compared with *c-Maf^fl/fl^* mice [*n* = 5 per group on TAM(10d); *c-Maf^fl/fl^*, *n* = 5 and *c-Maf^ΔTAM^*, *n* = 7 on STZ(12w) TAM(8w)]. (**J**) *Tgfβ* and (**K**) *Il-6* expression on STZ(12w) TAM(8w) (*c-Maf^fl/fl^*, *n* = 5; *c-Maf^ΔTAM^*, *n* = 7). Scale bars: 100 μm. **B**, **D**–**G**, and **I**–**K** were presented as the mean and the standard error of the mean (SEM). To assess whether differences between *c-Maf^ΔTAM^* and *c-Maf^fl/fl^* mice were statistically significant, a minimum of 3 biological replicates were analyzed using Welch’s *t* test, and a *P* value < 0.05 was considered significant. *P < 0.05, **P < 0.01, ***P < 0.001. White circles: *c-Maf^fl/fl^* groups, and black circles: *c-Maf^ΔTAM^* groups.

The observed decrease in glomerular albuminuria in *c-Maf*^ΔTAM^ mice (Figure 5) suggests that the loss of *c-Maf* may improve nephropathy and diabetic status. Interestingly, previous studies using diabetic *Sglt2*-knockout mice have shown that the loss of *Sglt2* can attenuate diabetic conditions, such as hyperglycemia and glomerular hyperfiltration, but not nephropathy (35). In contrast, diabetic mice treated with SGLT2 inhibitors reportedly showed improvement in the progression of diabetic nephropathy (40–43). Therefore, we investigated whether *c-Maf* deletion improves diabetic nephropathy by measuring periodic acid–methenamine silver–positive (PAMS-positive)and Sirius red–positive areas to examine the mesangial matrix index and fibrosis in the kidneys on STZ(12w) TAM(8w). PAMS staining was improved in *c-Maf*^ΔTAM^ mice compared with that in control mice (Figure 6, A and B, and Supplemental Figure 5, A and B), and Sirius red–positive areas were significantly decreased (Figure 6, C and D, and Supplemental Figure 5, C and D). In addition, the expression of *Col1a1*, *Col4a3*, and *fibronectin* genes was reduced in *c-Maf*^ΔTAM^ mice (Figure 6, E–G).

Since oxidative stress is a key molecular mechanism underlying diabetic nephropathy, including inflammation, fibrosis, and endothelial dysfunction (44, 45), we evaluated the levels of 8-hydroxydeoxyguanosine (8-OHdG), an oxidative stress marker, in the kidney tissue and urine, as well as the expression of *Il-6* and *Tgfβ* genes. Although there were no significant differences between the groups on TAM(10d), possibly because of the absence of excess oxidative stress, IHC and urine analyses revealed that 8-OHdG levels were decreased in *c-Maf*^ΔTAM^ mice on STZ(12w) TAM(8w) compared with those in *c-Maf^fl/fl^* mice (Figure 6, H and I). In addition, the expression of *Il-6* and *Tgfβ* genes was reduced in *c-Maf*^ΔTAM^ mice (Figure 6, J and K). Together, these data suggest that c-Maf deletion reduces oxidative stress under diabetic conditions, as well as the expression of *Il-6*, *Tgfβ*, *Col1a1*, *Col4a3*, and *fibronectin* mRNA, thereby suppressing the inflammatory reaction and fibrotic process.

Finally, we investigated the role of c-Maf in podocytes and immune cells in DKD and the immunological consequences of c-Maf deficiency (46, 47) by staining macrophages expressing c-Maf and WT1 in kidney podocytes on STZ(12w) TAM(8w) and examining the expression of *Il-10* and *Il-17* genes on TAM(10d) and STZ(12w) TAM(8w). Costaining for macrophages and c-Maf verified the infiltration of macrophages expressing c-Maf into the kidneys of both *c-Maf^fl/fl^* and *c-Maf*^ΔTAM^ mice (Supplemental Figure 5E). However, there were no significant differences in the numbers of WT1-positive nuclei or in the expression of *Il-10* and *Il-17* genes between the 2 groups (Supplemental Figure 5, F–K). RNA-Seq analysis revealed that the gene expression of *c-Mip*, which plays an important role in podocyte function and survival (48) and is strongly related to Wilms tumor protein (WT1), did not differ between *c-Maf^fl/fl^* and *c-Maf*^ΔTAM^ mice. Taken together, these results suggest that c-Maf loss exerts renoprotective effects against diabetic kidney injury and that these effects are not mediated through c-Maf loss in macrophages, WT1, c-Mip, IL-10, or IL-17.

In this study, we demonstrate that c-Maf directly regulates the expression of *Sglt2* and *Glut2* by binding to MARE sites and that *c-Maf* deletion attenuates STZ-induced diabetic nephropathy by improving diabetic conditions and reducing oxidative stress in the kidney.

## Discussion

The transcription factor c-Maf has been widely studied and has been reported to play a critical role in embryonic kidney development; however, the postnatal functions of c-Maf in adult kidneys remain unknown as *c-Maf*–null C57BL/6J mice exhibit embryonic lethality. In this study, we demonstrate that c-Maf directly regulates the expression of the glucose transporter genes, *Sglt2* and *Glut2*, thereby, lowering blood glucose levels under diabetic conditions. In addition, we demonstrate that *c-Maf* deletion can promote recovery from hyperglycemia and glomerular hyperfiltration by downregulating *Sglt2* and *Glut2*, which could be a key mechanism underlying improved kidney function. Moreover, *c-Maf*^ΔTAM^ mice showed reduced renal oxidative stress levels and *Col1a1*, *Col4a3*, *fibronectin*, *Il-6*, and *Tgfβ* mRNA expression, which may suppress the inflammatory reaction and fibrotic processes as the renal levels and expression of these genes are associated with mesangial cell proliferation, tubular atrophy, and kidney fibrosis (44, 45, 49–51). Therefore, these results suggest that c-Maf deletion improves diabetic conditions and has renoprotective effects.

However, the effects of whole-body *c-Maf* deletion on other organs that are involved in insulin and glucagon secretion and on several immune cells that are regulated by c-Maf (52, 53) must be considered as must be the limitations of using the STZ diabetic mouse model. The IHC staining in the pancreas and liver showed that there was no c-Maf expression in islet cells and hepatocytes. In addition, because there was no significant change in food intake between the *c-Maf^fl/fl^* and *c-Maf*^ΔTAM^ groups in STZ experiments, *c-Maf* deletion in the brain has less effect on Glut2, which plays a role in the regulation of food intake and body energy stores (54). Furthermore, from qPCR and IHC staining results, we found that factors, such as macrophages, IL-10, IL-17, WT1, and c-Mip, which are related to c-Maf regulation, may not be affected by *c-Maf* deficiency, at least in the kidney. Taken together with the fact that there were no significant changes in blood insulin, glucagon, and IPGTT in *c-Maf*^ΔTAM^ mice on TAM(10d) compared with that in *c-Maf^fl/fl^* mice, under conditions of this study, *c-Maf* deletion had almost no effect on Glut2 expression and insulin and glucagon secretion in the pancreas, liver, and brain and in several immune cells that are regulated by c-Maf.

Second, the STZ diabetic mouse model, although well accepted, has its limitations due to the cytotoxic effect of STZ on proximal tubular cells. In addition, STZ enters the cells through Glut2, and its cytotoxic effect also influences proximal tubular cells; hence, it has to be considered that similar albumin levels may reflect the damage caused by STZ to proximal tubules, regardless of the reduction in albumin transporter levels. In this study, to minimize the nonspecific toxicity of STZ on kidney proximal tubules in the C57BL/6 background mice, both multiple and low-dose injections of STZ were utilized (55, 56). By minimizing its toxicity, the effect on megalin function was minimized, resulting in the maintenance of albumin reabsorption. In fact, urinary albumin levels in *megalin*-knockout mice increased substantially, 2 weeks after TAM administration compared with the levels before TAM administration (8 weeks after STZ), whereas urinary albumin levels in *megalin^fl/fl^* mice increased before and after TAM administration. In other words, the maintenance of the megalin function resulted in the maintenance of albumin reabsorption, which suggests the amelioration of the amount of urinary albumin excretion in *c-Maf*^ΔTAM^ mice compared with that in *c-Maf^fl/fl^* mice.

In addition to the STZ diabetic mouse model, db/db mice of the C57BLKS/J strain are frequently used as a model of early to moderately advanced morphological changes in the kidney that are associated with diabetic nephropathy. Indeed, a proof of protective effects of *c-Maf* deletion not only in type 1 but also in type 2 diabetes using the mouse model can strengthen the notion of a close interaction between c-Maf and Sglt2. However, one of the main purposes of this study was to detect whether *c-Maf* deletion has protective effects on severe diabetic nephropathy caused by hyperglycemia. Numerous studies have reported that db/db mice show an increase in glomerular basement thickening, podocyte loss, moderate mesangial matrix expansion, oxidative stress, and inflammation in the kidneys, but they did not develop features of severe tubulointerstitial fibrosis. In contrast to db/db mice, although the STZ-induced C57BL/6 mouse model is milder than the models in other strains, these mice exhibit advanced renal pathological changes, including glomerular hypertrophy, excess oxidative stress, and inflammation, which are strongly implicated in the pathogenesis of diabetic nephropathy, resulting in severe tubulointerstitial fibrosis (57). For this reason, we selected STZ-induced C57BL/6 mice; in fact, *c-Maf^fl/fl^* mice showed severe tubulointerstitial fibrosis, and *c-Maf*^ΔTAM^ mice improved diabetic nephropathy as a result of *c-Maf* deletion. Therefore, we identified the high possibility of an important potentially novel function of c-Maf in adult kidneys and demonstrated that *c-Maf* deletion in the kidney exerts beneficial effects on diabetic nephropathy. In the future, db/db mice can be used as a useful model to observe the effects of *c-Maf*–deficient Sglt2 inhibition on multiple organs.

Although no previous studies have demonstrated that c-Maf is a major transcription factor regulating the expression of glucose transporters in kidney proximal tubules, we found that c-Maf directly regulates *Sglt2* and *Glut2*, which are responsible for the reabsorption of renally filtered glucose in proximal tubules. It has previously been reported that the transcription factors Hnf1α, Hnf3β, and Srebp1c link *Sglt2* expression (20, 22, 58, 59–61), while Hnf4α-mutant mice present with renal glycosuria due to the loss of Sglt2 (61, 62). Furthermore, Sp1 has been shown to promote Sglt1 and Sglt2 expression in the presence of Zn (63). Together, these studies indicate that these transcription factors play key roles in mammalian glucose homeostasis. Consistently, we found that the kidney glycosuria phenotype correlated with the downregulated expression of these transporters, indicating that c-Maf is a major transcription factor that regulates transporter expression in kidney proximal tubules. Interestingly, in the previous report, renal Glut2 deficiency reduced the expression of Sglt2 14 days after the third TAM injection via downregulation of the transcription factor HNF1α (20). In contrast, in this study, c-Maf deletion substantially decreased the expression of Sglt2 on only a day after the second TAM injection. Therefore, the time lag in the reduction of Sglt2 expression between previous studies and this study suggested direct regulation of Sglt2 by c-Maf. Furthermore, considering the lack of significant differences in Hnf1a expression, and the results of ChIP and Luc assays, we concluded that c-Maf directly controls the expression of Sglt2 and Glut2 in the kidney, respectively. In a future study, the cooperative functions of c-Maf and other transcription factors should be explored.

Our results also indicate a possibility that *c-Maf* is a potentially novel therapeutic target gene for diabetic nephropathy. SGLT2 blockades are considered the most influential method for improving diabetes. Here, we demonstrate that *c-Maf* deletion suppressed the development of not only hyperglycemia and hyperfiltration but also diabetic nephropathy, similar to the treatment with SGLT2 inhibitors in mice (40–43). In contrast, a previous study reported that *Sglt2*-knockout mice with diabetic nephropathy displayed attenuated hyperglycemia and glomerular hyperfiltration, but not kidney injury (35). The phenotypic differences between our study and the previous study using *Sglt2*-knockout mice suggest the presence of other factors as well as SGLT2 inhibitor treatment that attenuate diabetic nephropathy in *c-Maf*^ΔTAM^ mice.

After the discovery of SGLT2 inhibitors, Slc-mediated membrane transporters attracted increasing attention as new drug discovery targets. For example, prototypical loop diuretics, such as furosemide, bumetanide, and torsemide, can bind to chloride cotransporters (NKCCs: SLC12A1) and block ion transport directly and can, therefore, be used to treat heart failure, liver scarring, or kidney disease (64). Ezetimibe targets the Niemann–Pick C1–like 1 (NPC1L1: SLC65A2) protein, thereby reducing cholesterol absorption from the intestine (65). Recently, other Slc-mediated transporters have attracted attention as potential novel therapeutic targets. For instance, the inhibition of Glut2 (Slc2a2) dynamics has the potential to improve diabetes mellitus in mice (21), and Slc7A5, a glutamine antiporter, could be an attractive target for therapy-resistant, KRAS-mutant colorectal cancer in mice (66). In addition to Slc-mediated transporters, non–Slc-mediated transmembrane transporters have potential as therapeutic agents. Tolvaptan, a competitive vasopressin receptor 2 antagonist, has been used to treat hyponatremia associated with congestive heart failure, cirrhosis, and the syndrome of inappropriate antidiuretic hormone (67). In this study, *c-Maf*^ΔTAM^ mice excreted higher urinary albumin levels on TAM(10d) than did *c-Maf^fl/fl^* mice due to the downregulation of the expression of megalin and cubilin genes. Previous studies have reported that albumin reabsorption in the proximal tubules promotes renal damage and that proximal tubular proteinuria reduces the tubular burden (68–70). Therefore, it is conceivable that various Slc- and non–Slc-mediated transmembrane transporters could be potential therapeutic agents for various diseases. Indeed, we demonstrate a strong relationship between c-Maf and Slc-mediated transporters, such as glucose transporters and amino acid transporters (Figure 2A), indicating that c-Maf may play an important role in controlling not only the transporters that are known therapeutic agents but also other unknown transporters. In fact, a previous study reported that by suppressing the cellular excess of amino acids, CB1R maintains the normal activity of mTORC1, which may help ameliorate DKD (22). Therefore, the presence of amino acid transporters as a drug target controlled by c-Maf can be an advantage of c-Maf inhibition/deletion over SGLT2 inhibition. Although, in the future, it will be important and necessary to clarify the relationship between c-Maf and these transporters, c-Maf has potential as a comprehensive therapeutic target in the treatment of not only diabetic nephropathy but also other diseases.

The limitations of targeting c-Maf specifically in the kidney must be considered. Currently, treatment of CKD requires drugs, such as hormones and nonsteroidal antiinflammatory drugs, to be delivered at high concentrations into the kidney, which results in adverse systemic reactions because of the nondiscriminatory distribution of the drugs to sites other than the kidneys (71). Therefore, this can presently be a limitation of targeting c-Maf, specifically in the kidney. However, the concurrent rapid development of nanotechnology and macromolecular carrier technology and emerging preclinical evidence have greatly facilitated advancements in kidney-targeted drug delivery systems. These systems have offered the possibility to plan a cell-targeted administration of new and traditional drugs (72, 73). There are several hurdles, such as solubility, toxicity, and suboptimal renal targeting, which slow the transition toward clinical applications. While renal drug delivery technologies are still in the preclinical stage, the efficacy of a wide array of therapeutics delivered using the diverse drug delivery platforms has been demonstrated by in vitro studies and through in vivo testing in animal models. Therefore, emerging preclinical evidence supports the feasibility of a drug delivery technology for the treatment of kidney diseases with different etiologies and has been offering novel platforms and tools that foster opportunities for the gradual development of new treatments (74).

In conclusion, this study elucidates potentially unique mechanisms of *c-Maf* gene regulation as well as the renoprotective effects of *c-Maf* deletion under diabetic conditions through the regulation of *Sglt2* and *Glut2* in the kidneys. Considering the strong association between c-Maf and these novel therapeutic target genes for diabetes and diabetic nephropathy, our research contributes toward novel advances in basic and clinical investigation.

## Methods

### Animals.

All animals were maintained under specific pathogen–free conditions at the Laboratory Animal Resource Center of the University of Tsukuba, Ibaraki, Japan. All experiments were performed according to the guidelines of the Care and Use of Laboratory Animal Resource Center at the University of Tsukuba and were approved by its Institutional Review Board. *CAG-Cre-ER*^TM^ transgenic mice were purchased from the Jackson Laboratory to generate *c-Maf*–conditional knockout mice (*c-Maf^fl/fl^*; *CAG-Cre-ER*^TM^) (75). *c-Maf^fl/fl^* mice (*c-Maf^fl/fl^*) containing *loxP* sites were generated as reported previously (11). To activate the Cre recombination system, 8-week-old *c-Maf^fl/fl^*; *CAG-Cre-ER*^TM^ mice were injected intraperitoneally with 75 mg TAM/kg for 5 consecutive days. These mice are herein referred to as “*c-Maf*^ΔTAM^” mice. *c-Maf^fl/fl^* control mice were also treated with TAM. TAM (T5648-1G; MilliporeSigma) was first dissolved in ethanol and then mixed with corn oil as described previously (76). In addition, 6-week-old mice with the β-galactosidase (*LacZ*) gene inserted into the *c-Maf* locus were used to visualize c-Maf accumulation in the kidneys (5). Type 1 diabetes was induced in the mice through consecutive intraperitoneal injections with STZ (195-15154; FUJIFILM Wako Pure Chemical Corporation; 50 mg/kg body weight), freshly prepared in 0.05 M sodium citrate buffer (pH 4.5), for 5 days after 5–6 hours of fasting. Blood glucose levels were measured 3 weeks after the last STZ injection to identify mice with levels > 300 mg/dL. Mice with this level were considered diabetic and were used in subsequent experiments. Food intake was measured for 24 hours. The detailed experimental protocol is provided in Supplemental Figure 4A.

### Urine and serum analysis.

Mice were housed in metabolic cages for 24 hours, and urine samples were then collected from each mouse. The urine samples were placed on ice during collection and then stored at −30°C. Urinary levels of 8-OHdG and other urinary components were evaluated by the Japan Institute for the Control of Aging, Nikken SEIL, and the Oriental Yeast Corporation, respectively. Amino acids were analyzed using an Automatic Amino Acid Analyzer (JEOL JLC-500/V2) by Tamaki Hirose at Chemical Analysis Division, Research Facility Center for Science and Technology, University of Tsukuba. Serum amino acid levels were evaluated by the Tohoku Medical Megabank Organization, Tohoku University, Sendai, Japan. Water intake was measured using a specific bottle (Shinfactory). Serum samples were stored at about 25°C for approximately 15 minutes followed by 2–4 hours on ice, centrifuged at 1,450*g* for 10 minutes at 15°C, and frozen at −30°C. Serum components were evaluated by the Oriental Yeast Corporation. Serum insulin and glucagon levels were examined using an Ultra-Sensitive Mouse Insulin ELISA Kit (M1108; Morinaga) and a Glucagon ELISA Kit (10-1281-01; Mercodia), respectively.

### IPGTT.

After 10–12 hours of fasting, blood samples were collected from the tail vein, and basal blood glucose levels were measured at 0 minutes. The mice were then intraperitoneally injected with a glucose solution (20% glucose and 2% of glucose/kg body mass) using an Otsuka Glucose Injection 50% (M7I73) diluted in saline, and blood glucose levels were measured, 15, 30, 60, and 120 minutes after injection, using the Medisafe FIT Blood Glucose Meter (TERUMO).

### qPCR.

Total RNA was extracted from the kidneys of *c-Maf*^ΔTAM^ and *c-Maf^fl/fl^* mice using the ISOGEN reagent (311-02501; Nippon Gene). cDNA was synthesized using a QuantiTect reverse transcription kit (205313; QIAGEN) according to the manufacturer’s protocol. qPCR was performed using a Thermal Cycler Dice real-time system (TaKaRa) with THUNDERBIRD SYBR qPCR Mix (QPS-201; Toyobo). The primers listed in Supplemental Table 3 were used to amplify and detect the target genes. The expression of target genes was normalized to that of *Hprt*.

### Histopathological analysis.

IHC analysis was performed as described previously (77), with some modifications, in 3-, 6-, or 8-week-old mice to detect c-Maf, Sglt2 and Glut2, β-galactosidase, 8-OHdG, albumin, macrophages, and WT1. Briefly, kidneys were collected from *c-Maf*^ΔTAM^ and *c-Maf^fl/fl^* mice, fixed in 10% Formalin Neutral Buffer Solution (133-10311; FUJIFILM Wako Pure Chemical Corporation) at 4°C for 24 hours, embedded in paraffin, cut into 3 μm–thick sections, and placed on slides. After removal of paraffin, the tissue sections were washed with phosphate-buffered saline (PBS), then subjected to antigen retrieval (S1699; Dako) for 1 hour at 90°C for c-Maf, Sglt2, Glut2, β-galactosidase, 8-OHdG, albumin, macrophages, and WT1. Approximately 30 minutes after being cooled to about 25°C, the samples were washed with PBS and permeabilized with 0.3% Triton X-100/PBS to detect β-galactosidase; this process was skipped for c-Maf, Sglt2, Glut2, albumin, macrophage, and WT1 detection. The samples were then blocked for 1 hour in PBS with 5% bovine serum albumin (BSA) and 10% goat serum, then incubated for 40 hours at 4°C with the following primary antibodies: anti–c-Maf rabbit monoclonal antibody (1:200; M00654; Boster Biological Technology), anti-Sglt2 (1:200; ab85626; Abcam), anti-Glut2 (1:200; 600-401-GN3; ROCKLAND), rabbit anti–β-galactosidase (1:200; 4600-1409; Biogenesis), anti-albumin rabbit polyclonal antibodies (1:200; GTX102419; GeneTex), anti-macrophage rat monoclonal antibodies (RM0029-11H3) (1:200; ab56297; Abcam), and anti-WT1 rabbit monoclonal antibodies [CAN-R9(IHC)-56-2] (1:300; ab89901; Abcam). To detect β-galactosidase, c-Maf, Sglt2, Glut2, macrophages, and WT1, samples were washed with PBS, then stained with Histofine Simple Stain MAX-PO (414341; Nichirei Biosciences), and signals were amplified using a Histofine DAB substrate kit (425011; Nichirei Biosciences). The sections were mounted with Fluoromount (Diagnostic BioSystems). To detect 8-OHdG, samples were deparaffinized and subjected to antigen retrieval for 10 minutes at 121°C. Approximately 30 minutes after cooling at 23°C–26°C, the samples were blocked for 1 hour in PBS with 5.0% BSA and incubated for 40 hours at 4°C with anti–8-OHdG monoclonal antibodies (N45.1; 1:200; JaICA, Nikken SEIL). After washing with PBS, samples were stained with Histofine Simple Stain AP(M) (414251; Nichirei Biosciences) for signal amplification, and color signals were amplified using a Histofine Alkaline Phosphatase Detection Kit (415261; Nichirei Biosciences). All images were acquired using a BIOREVO BZ-9000 microscope (Keyence). For electron microscopy, tissues were fixed with 2.5% glutaraldehyde in PBS. Standard methods were used to perform transmission electron microscopy.

### Measurement of PAMS- and Sirius red–positive area and the number of WT1-positive nuclei.

The PAMS-positive area was detected as described previously (78), with some modifications. The degree of mesangial matrix expansion, tubulointerstitial fibrogenesis, and the number of WT1-positive nuclei were assessed by measuring the PAMS-positive and mesangial Sirius red–positive areas and by performing IHC staining for WT1 in the kidneys. PAMS-stained glomeruli (20–25 fields at 40× original magnification), Sirius red–stained interstitial lesions (20–27 fields at 20× original magnification), and the number of WT1-positive nuclei (15–20 fields at 40× original magnification) were evaluated in each mouse using a BIOREVO BZ-9000 microscope. Glomerular size (tuft area) was measured by tracing the tuft (red color), while the mesangial matrix area was defined as the PAMS-positive area (green color within the tuft area). The mesangial matrix index represents the ratio of the mesangial matrix area to the tuft area. Tubulointerstitial lesions, defined as Sirius red–positive areas (blue color), were measured to detect fibrogenesis and expressed as a percentage of the total area. The number of WT1-positive nuclei (green color) was counted within the tuft area and divided by the area of the glomerulus.

### ChIP assay.

The ChIP Assay Kit protocol (17-295; MilliporeSigma) was modified based on a previous study (79). Briefly, kidneys extracted from *c-Maf*^ΔTAM^ and *c-Maf^fl/fl^* mice were fixed in 1% paraformaldehyde at 4°C for 24 hours and stored at −80°C until use. The samples were incubated for 10 minutes in ChIP lysis buffer (50 mM Tris-HCl [pH 7.9], 10 mM ethylenediaminetetraacetic acid [EDTA], 0.1% sodium dodecyl sulfate [SDS], and 1 mM phenylmethylsulfonyl fluoride [PMSF]) and sonicated to shear the chromatin into 500–1,000 bp DNA fragments. After centrifugation at 15,000 rpm for 10 minutes at 4°C, the supernatant was collected and incubated overnight at 4°C with rotation in ChIP dilution buffer (16.7 mM Tris-HCl [pH 7.9], 16.7 mM NaCl, 1.2 mM EDTA, 1.1% Triton X-100, 0.01% SDS, and 1.1 mM PMSF) and Protein A/BSA/ssDNA (Protein A beads, 10 mM Tris-HCl [pH 7.9], 1.0 mM EDTA, 0.1% Triton X-100, 0.05% NaN_3_, 0.5 mg/mL BSA, and 0.2 mg/mL salmon sperm DNA). Samples were then centrifuged at 730*g* for 5 minutes at 4°C and the supernatant was collected. Two-thirds of the lysates were incubated with anti-H3 antibodies (ab1791; Abcam), c-Maf antibodies (A300-613A; Bethyl), and normal rabbit IgG (12-370; MilliporeSigma) with rotation at 4°C overnight. One-third of the lysates were used as input samples. After incubation, Protein A/BSA/ssDNA was added, and the samples were incubated at 4°C for 1 hour. After centrifugation at 730*g* for 5 minutes at 4°C, the supernatant was discarded, and the antibody–protein–DNA complexes were washed twice with a high-salt wash buffer (20 mM Tris-HCl [pH 7.9], 500 mM NaCl, 2 mM EDTA, 1% Triton X-100, and 0.1% SDS), once with LiCl wash buffer (10 mM Tris-HCl [pH 7.9], 250 mM LiCl, 1 mM EDTA, 1% NP-40, and 1% sodium deoxycholate), and twice with Tris-EDTA (pH 7.9). The samples were then incubated in an elution buffer (100 mM NaHCO_3_, 1% SDS, and 10 mM dithiothreitol) at about 25°C for 15 minutes, then centrifuged at 730*g* for 1 minute at about 25°C, and the supernatant was collected. This elution process was repeated in duplicate. The samples and input samples were incubated at 65°C overnight with an elution buffer and 5 M NaCl, then treated with 1 M Tris-HCl (pH 6.2), 0.5 M EDTA, and 20 μg/mL Proteinase K (29442-85; Nacalai Tesque) at 37°C for 1 hour, and then DNA was extracted using phenol:chloroform:isoamyl alcohol and precipitated using ethanol. DNA fragments were subjected to qPCR using KOD SYBR qPCR Mix (QKD-201; Toyobo) with the specific primer sets listed in Supplemental Table 1.

### Dual-luciferase assays and site-directed mutagenesis.

*Sglt2* and *Glut2* sequences including the MARE (−1,019 to −53 bp and −303 to 287 from transcription start site, respectively) were generated using PCR with the primers listed in Supplemental Table 3. These products were digested using XhoI and KpnI and inserted upstream of luc2CP in the pGL4.28 vector. Clones were confirmed using sequencing. Site-directed mutagenesis of *Sglt2* and *Glut2* was performed using a KOD-Plus- Mutagenesis Kit (SMK-101; Toyobo) with the primers listed in Supplemental Table 3. For the luciferase assay, HEK293T cells purchased from ATCC were seeded in 24-well plates, incubated for 17–24 hours, and then cotransfected with pGL4.28 containing the inserted *Sglt2* and *Glut2* sequences (100 ng/μL) and PEFX containing the inserted *c-Maf* sequence (0, 10, 30, or 90 ng/μL). Renilla luciferase signals were normalized to pRL-TK (50 ng/μL for *Sglt2*, *Glut2*), and GFP was used to confirm successful transfection. A total of 300 ng of vectors were added to each well. These experiments were performed 3 times in triplicate. Dual-luciferase assays were performed using the Dual-Luciferase Reporter Assay System (E1910; Promega). After transfection for about 24 hours for *Sglt2* and *Glut2*, the cells were lysed, and 20 μL cell lysate was mixed with 100 μL Luciferase Assay Reagent II. Samples were measured using a GloMax 20/20 Luminometer (E5311; Promega) and again after Stop & Glo reagent (100 μL) was added to each sample.

### RNA-Seq transcriptome analysis.

Total RNA was isolated using the TRIzol reagent (Thermo Fisher Scientific). RNA quality was examined using an RNA 6000 Pico Kit (Agilent). A total of 50 ng RNA was used for the preparation of the RNA-Seq library using an NEB Next rRNA Depletion Kit and an NEB Next Ultra Directional RNA Library Prep Kit (New England Biolabs). The 2 × 36 base paired-end sequencing was performed by Tsukuba i-Laboratory LLP using NextSeq 500 technology (Illumina). FASTQ files were imported into the CLC Genomics Workbench (Version 10.1.1; QIAGEN). Sequence reads were mapped to the mouse reference genome (mm10). Gene expression values were calculated using the quantile method. Gene expression was compared between *c-Maf^fl/fl^* control mice and *c-Maf*^ΔTAM^ mice using the Empirical Analysis DGE tool (edgeR test) in the CLC Genomics Workbench. False discovery rate (FDR) adjustments (80) were applied to correct for multiple testing, and DEGs were identified using an FDR cutoff of 0.05. The volcano plots were generated using a custom R script. Pathway analysis was performed using Metascape (81). All custom scripts for RNA-Seq analysis are available at https://github.com/akikuno/Maf_cKO_Kidney (commit ID 27dca2d). RNA-Seq data in this paper are available in NCBI Gene Expression Omnibus, accession number GSE199916. Heatmaps were generated using Python3 and Morpheus (https://software.broadinstitute.org/morpheus/).

### Statistics.

All data were presented as the mean and the standard error of the mean. To assess whether differences between *c-Maf*^ΔTAM^ and *c-Maf^fl/fl^* mice were statistically significant, a minimum of 3 biological replicates were analyzed using Welch’s *t* test, and a *P* < 0.05 was considered significant. Holm-Šídák corrections were applied for multiple statistical tests in Figure 4, D and H; Figure 5A; and Supplemental Figure 4J.

### Study approval.

All experiments were performed according to the guidelines of the Care and Use of Laboratory Animal Resource Center at the University of Tsukuba and were approved by its Institutional Review Board.

## Author contributions

ST supervised the study. MF designed the experiments. MF and MO performed the experiments. MF and AK detected MARE sites on *Sglt2* and *Glut2*. MF analyzed data and drafted the manuscript. MF, TH, SI, and AK analyzed RNA-Seq data. SK analyzed serum amino acids. MF, NM, TH, SI, MO, AK, SK, KY, and ST edited and revised the manuscript. All authors interpreted the experimental results and approved the final version of the manuscript.

## Supplementary Material

Supplemental data

## Version 1 02/14/2023

In-Press Preview

## Version 2 03/22/2023

Electronic publication
