# Supplementary material for: Transcription factor c-Maf deletion improves streptozotocin-induced diabetic nephropathy by directly regulating Sglt2 and Glut2
Source: JCI Insight. 2023 Mar 22;8(6):e163306. doi: 10.1172/jci.insight.163306 (PMC10070115; doi:10.1172/jci.insight.163306)
Supplement: Supplemental data [file jciinsight-8-163306-s028.pdf]

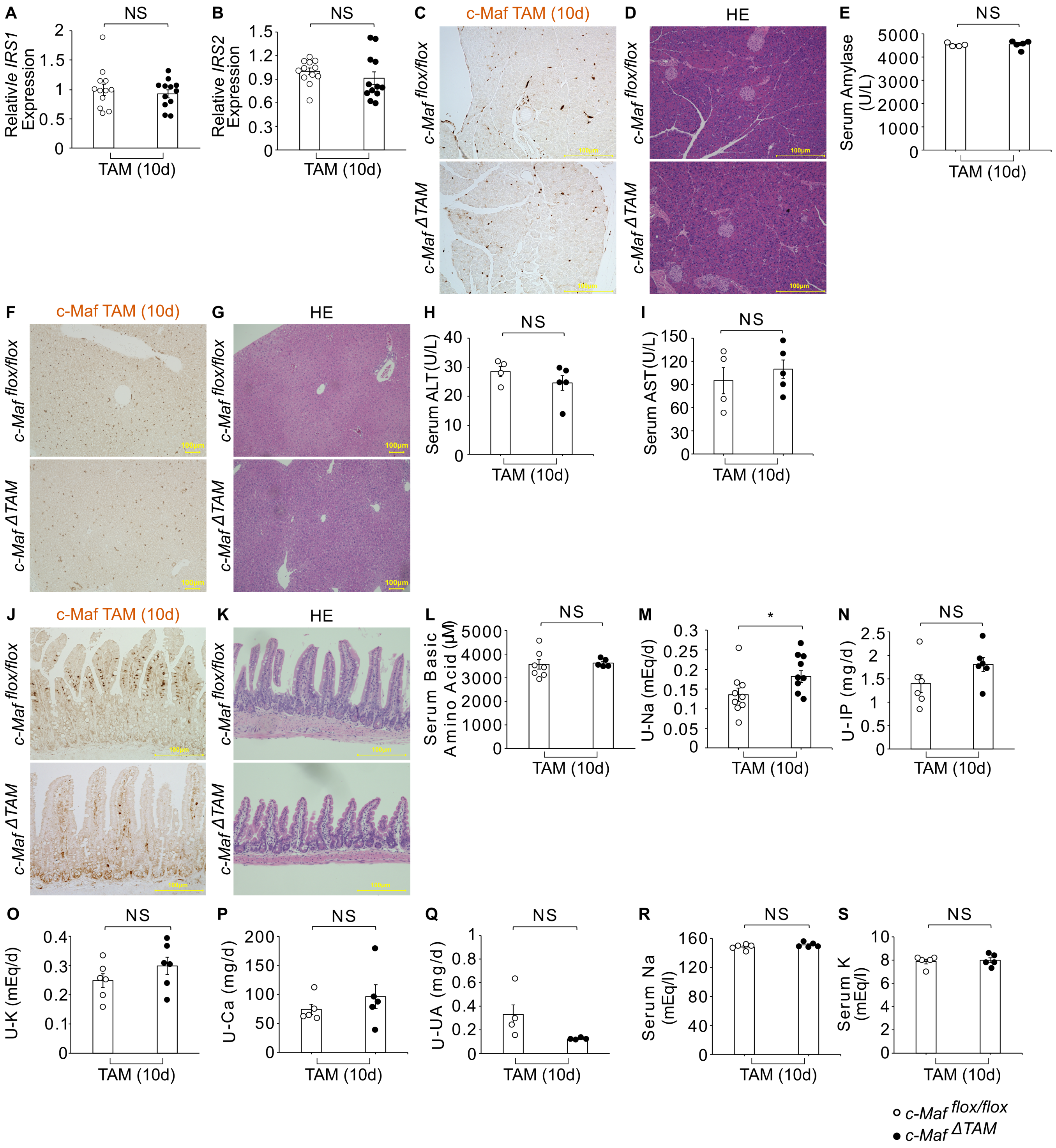

A
Relative IRS1 Expression
NS
TAM (10d)
B
Relative IRS2 Expression
NS
TAM (10d)
C
c-Maf TAM (10d)
c-Maf flox/flox
c-Maf ΔTAM
D
HE
100μm
E
Serum Amylase (U/L)
NS
TAM (10d)
F
c-Maf TAM (10d)
G
HE
H
Serum ALT(U/L)
NS
TAM (10d)
I
Serum AST(U/L)
NS
TAM (10d)
J
c-Maf TAM (10d)
K
HE
L
Serum Basic Amino Acid (μM)
NS
TAM (10d)
M
U-Na (mEq/d)
*
TAM (10d)
N
U-IP (mg/d)
NS
TAM (10d)
O
U-K (mEq/d)
NS
TAM (10d)
P
U-Ca (mg/d)
NS
TAM (10d)
Q
U-UA (mg/d)
NS
TAM (10d)
R
Serum Na (mEq/l)
NS
TAM (10d)
S
Serum K (mEq/l)
NS
TAM (10d)
○ c-Maf flox/flox
● c-Maf ΔTAM

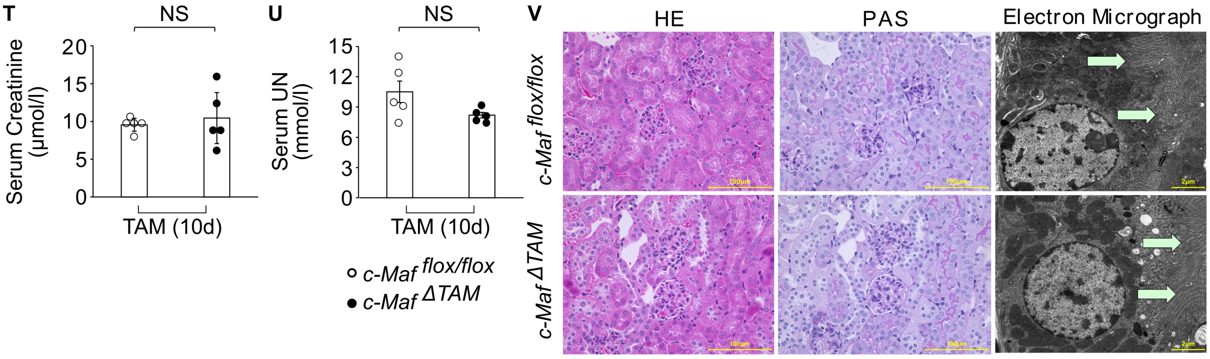


**Supplemental Figure 1. *c-Maf*$^{\Delta TAM}$ and *c-Maf*$^{flox/flox}$ mice showed comparable pancreas, liver, small intestine and kidney function and structure, but decreased albumin reabsorption in the proximal tubules and excess basic amino acid excretion.** (**A**) *IRS1* and (**B**) *IRS2* mRNA levels were analyzed in kidney tissues extracted from *c-Maf*$^{flox/flox}$ and *c-Maf*$^{\Delta TAM}$ mice using qPCR (n = 12 per group). (**C**) c-Maf expression around the exocrine cells, such as the macrophages and interstitial area. (**D**) No morphological changes in the pancreas and (**E**) no significant difference in amylase levels in the serum under the feeding conditions in *c-Maf*$^{\Delta TAM}$ mice compared with those in *c-Maf*$^{flox/flox}$ mice on TAM(10d). (**E**) Serum Amylase levels did not significantly differ between the two groups. (**F**) c-Maf expression around the hepatic cells, such as sinusoidal cells and macrophages. (**G**) No morphological changes in the liver and no significant difference in (**H**) ALT and (**I**) AST levels in the serum on TAM(10d). (*c-Maf*$^{flox/flox}$, n = 4; *c-Maf*$^{\Delta TAM}$, n = 5). (**J**) c-Maf expression and (**K**) no morphological changes in the small intestine. [C, D, F, G, J, K: *n* = 4 per group. E, H, I: *c-Maf*$^{flox/flox}$, n = 4; *c-Maf*$^{\Delta TAM}$, n = 5.] An almost identical metabolic phenotype was documented in *c-Maf*$^{flox/flox}$ and *c-Maf*$^{\Delta TAM}$ kidneys. (**L**) Serum basic amino acid, (**M**) Urine Na, (**N**) Urine IP , (**O**) Urine K, (**P**) Urine Ca, (**Q**) Urine UA, (**R**) Serum Na and (**S**) Serum K levels did not significantly differ between the two groups [L: *c-Maf*$^{flox/flox}$, n = 7; *c-Maf*$^{\Delta TAM}$, n = 5. M, N, O: n = 5. Q: n = 4. P, R, S: n = 5]. (**T**) Serum creatinine and (**U**) serum UN levels did not differ significantly between the two groups (n = 5 per group). The structures of glomeruli, proximal tubules, and brush border membranes in *c-Maf*$^{flox/flox}$ and *c-Maf*$^{\Delta TAM}$ were analyzed using electron microscopy in (**V**) HE-stained and PAS-stained kidney tissues (n = 4 per group). Green arrows indicate the brush border membrane. Scale bars: 100 μm for IHC, HE, and PAS staining, and 2 μm for electron microscopy images. Supplemental Figure 1A, B, E, H, I, L–U were presented as the mean and the standard error of the mean (SEM). To assess whether differences between *c-Maf*$^{\Delta TAM}$ and *c-Maf*$^{flox/flox}$ mice were statistically significant, a minimum of three biological replicates were analyzed using Welch's t test, and a P-value < 0.05 was considered significant. NS: not significant, **P* < 0.05, ****P* < 0.001.White circles- *c-Maf*$^{flox/flox}$ groups, and black circles- *c-Maf*$^{\Delta TAM}$ groups.

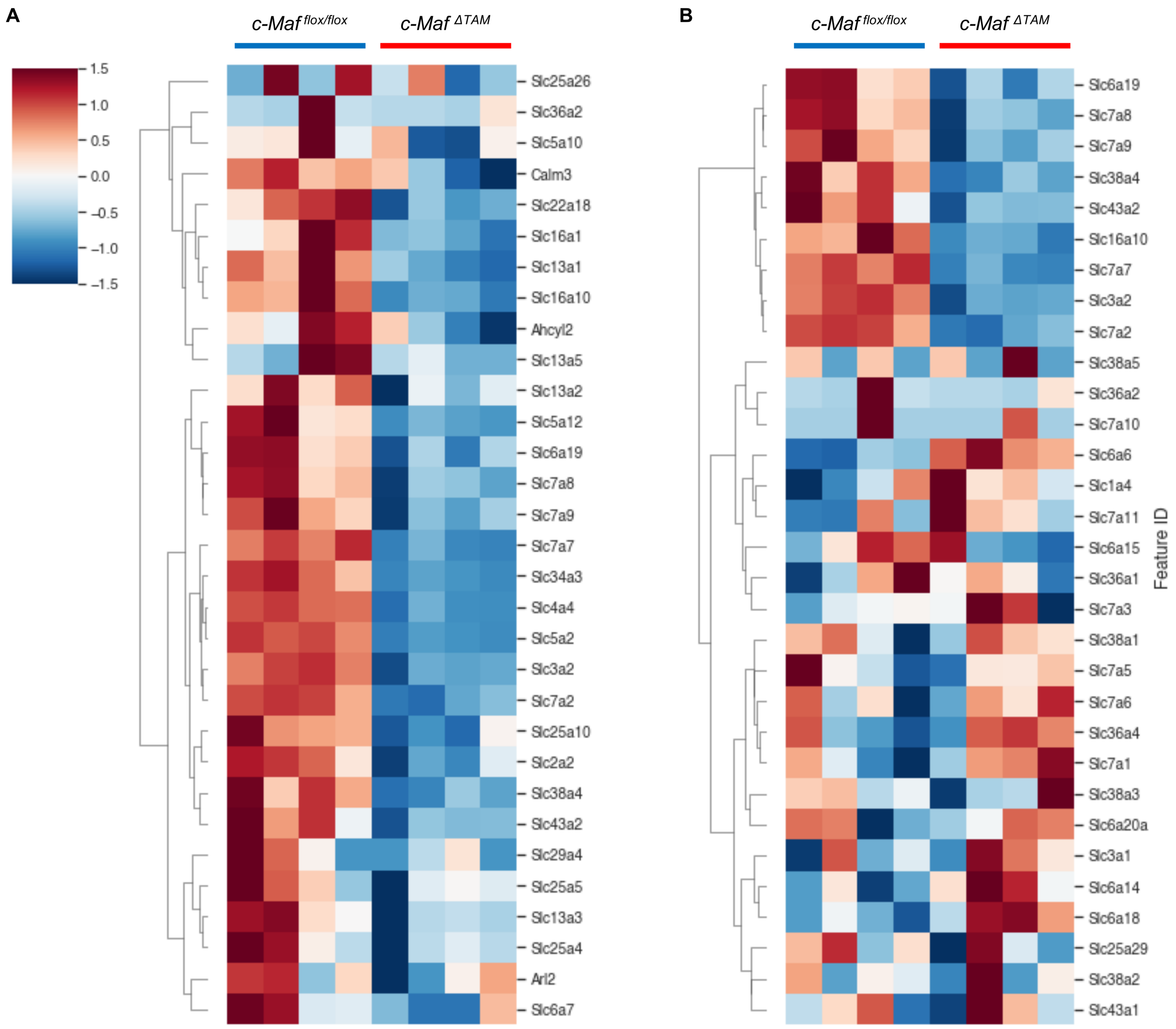


**Supplemental Figure 2. RNA-seq analysis of the relationship between c-Maf and renal features.** (**A**) Heatmap of claster2 expression, which clearly differed between *c-Maf*$^{\Delta TAM}$ and *c-Maf*$^{flox/flox}$ mice. (**B**) Heatmap of gene expression specific to the Metabolism of amino acids and derivatives ($n$ = 4 per group).

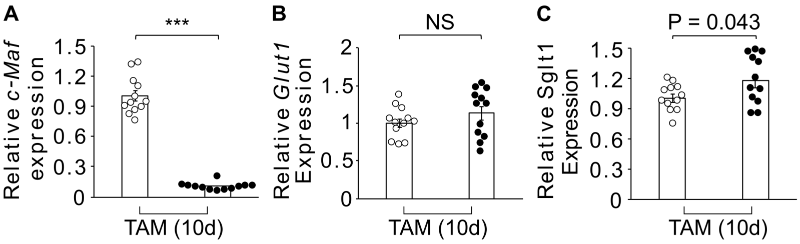


**Supplemental Figure 3. qPCR results showing a significant decrease in *c-Maf* expression, comparable *Glut1* expression, and an increase in *Sglt1* expression in *c-Maf*$^{\Delta TAM}$ mice compared to those in *c-Maf*$^{flox/flox}$ mice.** The renal mRNA levels of (**A**) *c-Maf* were significantly lower, (**B**) *Glut1* levels did not change in both groups, and (**C**) *Sglt1* was upregulated to compensate for reduced *Sglt2* in *c-Maf*$^{\Delta TAM}$ compared to *c-Maf*$^{flox/flox}$ group at TAM(10d). [A, B,C: n=12 per group]. Supplemental Figure 3A–C were presented as the mean and the standard error of the mean (SEM). To assess whether differences between *c-Maf*$^{\Delta TAM}$ and *c-Maf*$^{flox/flox}$ mice were statistically significant, a minimum of three biological replicates were analyzed using Welch's t test, and a P-value < 0.05 was considered significant. NS: Not Significant, and ***$P < 0.001$. White circles- *c-Maf*$^{flox/flox}$ groups, and black circles- *c-Maf*$^{\Delta TAM}$ groups.

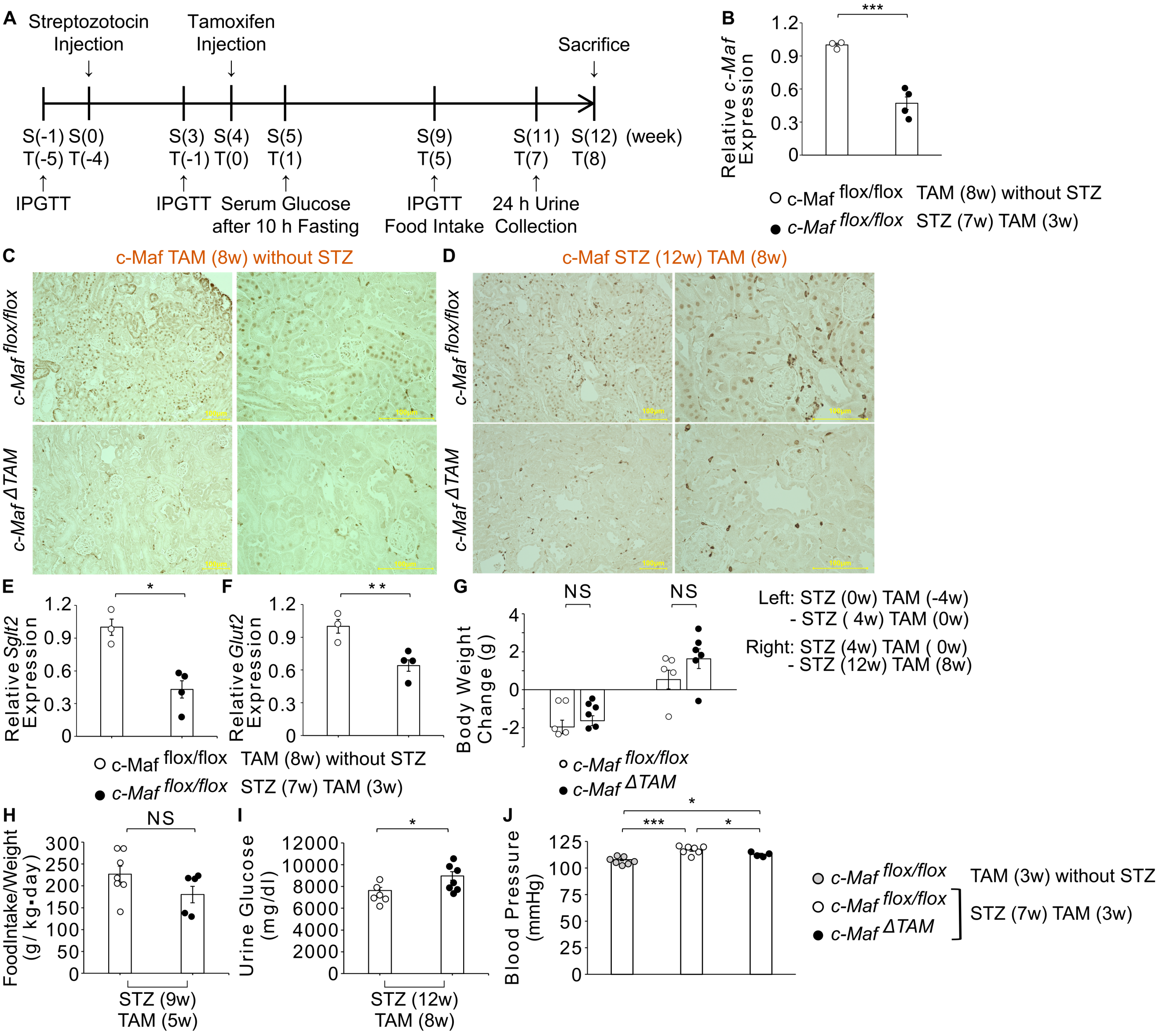


**Supplemental Figure 4. Schedule of streptozotocin and tamoxifen administration, detection of c-Maf expression and localization, and body weight, food intake, urinary glucose levels, and blood pressure in *c-Maf$^{\Delta TAM}$* and *c-Maf$^{flox/flox}$* mice under diabetic and/or non-diabetic conditions.** (**A**) S, streptozotocin (STZ); T, tamoxifen (TAM); IPGTT, intraperitoneal glucose tolerance test. Numbers in parentheses indicate the number of weeks after STZ or TAM administration. Under diabetic conditions, (**B**) *c-Maf* gene expression was significantly lower in the kidneys of *c-Maf$^{flox/flox}$* mice on STZ(7w) TAM(3w) compared to TAM(8w) without STZ. There was no change in c-Maf localization between these groups on (**C**) TAM(8w) without STZ or (**D**) STZ(12w) TAM(8w). (**E**) *Sglt2* and (**F**) Glut2 gene expression was significantly lower in the kidneys of *c-Maf$^{flox/flox}$* mice on STZ(7w) TAM(3w) compared to TAM(8w) without STZ. [B, E, F: $n$ = 3 on TAM(8w) without STZ; $n$ = 4 on STZ(7w) TAM(3w). C, D: $n$ = 3 per group.] (**G**) Body weight did not significantly differ between the groups on STZ(4w) TAM(0w). A tendency for increased body weight was observed in *c-Maf$^{\Delta TAM}$* mice at STZ(12w) TAM(8w) compared to control mice (*c-Maf$^{flox/flox}$*, $n$ = 5; *c-Maf$^{\Delta TAM}$*, $n$ = 6). (**H**) Food intake did not significantly differ between the groups on STZ(9w) TAM(5w) (*c-Maf$^{flox/flox}$*, $n$ = 7; *c-Maf$^{\Delta TAM}$*, $n$ = 5). (**I**) Urinary glucose levels were higher in *c-Maf$^{\Delta TAM}$* mice than in control mice on STZ(12w) TAM(8w) (*c-Maf$^{flox/flox}$*, $n$ = 6; *c-Maf$^{\Delta TAM}$*, $n$ = 7). (**J**) Blood pressure was lower in *c-Maf$^{\Delta TAM}$* mice than in control mice (*c-Maf$^{flox/flox}$* on TAM(3w) without STZ and STZ(7w) TAM(3w), $n$ = 7; *c-Maf$^{\Delta TAM}$* on STZ(7w) TAM(3w), $n$ = 4). Scale bars: 100 µm. Supplemental Figure 4B, E–J were presented as the mean and the standard error of the mean (SEM). To assess whether differences between *c-Maf$^{\Delta TAM}$* and *c-Maf$^{flox/flox}$* mice were statistically significant, a minimum of three biological replicates were analyzed using Welch's t test, and a P-value < 0.05 was considered significant. Holm corrections were applied for multiple statistical tests in Supplemental Figure 4J. NS: not significant, *$P$ < 0.05, ***$P$ < 0.001. White circles- *c-Maf$^{flox/flox}$* groups, and black circles- *c-Maf$^{\Delta TAM}$* groups.

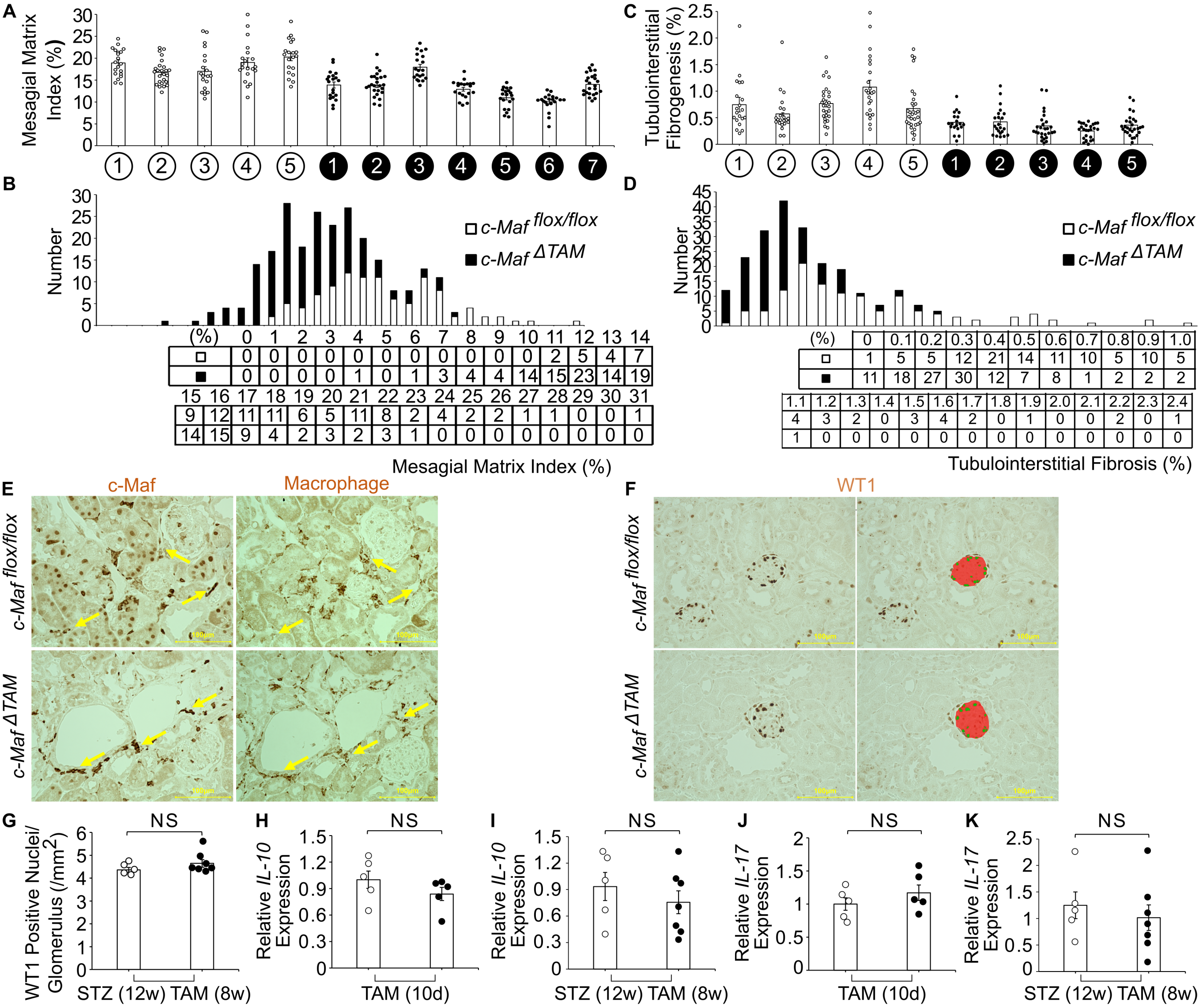


**Supplemental Figure 5. Morphometric analysis of glomerular and fibrosis, immunohistochemical staining for macrophage with c-Maf and WT1 expression, and qPCR analysis of *Il-10* and *Il-17*.** (**A**) Mesangial matrix indexes, (**B**) their frequency distribution, (**C**) ratio of tubulointerstitial fibrogenesis, and (**D**) their frequency distribution for all samples in both groups are displayed. [A, B: *c-Maf$^{flox/flox}$*, n=5; *c-Maf$^{\Delta TAM}$*, n =7. C, D: n =5 per both groups.] (**E**) Co-staining of macrophage with c-Maf showed infiltration of the macrophages expressing c-Maf into the kidneys in both groups. [E: n =3 per both groups.] (**F**) IHC staining for WT1, and (**G**) the quantitative evaluation showing no significant difference in the number of WT1 positive nuclei per glomerular size. [F: n =3 per both groups. G: *c-Maf$^{flox/flox}$*, n=5; *c-Maf$^{\Delta TAM}$*, n =7.] Comparable expression level of (**H** and **I**) *Il-10* and (**J** and **K**) *Il-17* on TAM(10d) and STZ (12w) TAM (8w), respectively. [I, K *c-Maf$^{flox/flox}$*, n=5; *c-Maf$^{\Delta TAM}$*, n =7. H, J: n=5 per both groups]. Supplemental Figure 5G–K were presented as the mean and the standard error of the mean (SEM). To assess whether differences between *c-Maf$^{\Delta TAM}$* and *c-Maf$^{flox/flox}$* mice were statistically significant, a minimum of three biological replicates were analyzed using Welch's t test, and a P-value < 0.05 was considered significant. NS: not significant. White circles- *c-Maf$^{flox/flox}$* groups, and black circles- *c-Maf$^{\Delta TAM}$* groups.

**Supplemental Table 1.** Primer sequences for ChIP assays

| Gene | Forward/reverse primer sequence | |
|---|---|---|
| *c-Maf* | Forward | 5′ -CTGCCGCTTCAAGAGGGTGCAGC-3′ |
| | Reverse | 5′ -TCGCGTTCACACTCACATG-3′ |
| *Sglt2* | Forward | 5′ -CCCAGGAAGGAGTGCTCTTG-3′ |
| | Reverse | 5′ -GACAAGTCCCCCAGGTCTCA-3′ |
| *Sglt2* (negative control) | Forward | 5′ -GGTCACCAGGCAAGTTAGGC-3′ |
| | Reverse | 5′ -CCCCAGACTGCACCTCCTTA-3′ |
| *Glut2* | Forward | 5′ -TGGGGTAAAGGGTGTATTGATTG-3′ |
| | Reverse | 5′ -TGGAATTGTCCTCTTAATCCAGGT-3′ |
| *Glut2* (negative control) | Forward | 5′ -TCGTTAGGAATGAGGTGACACCA-3′ |
| | Reverse | 5′ -CAGGAAAATGAAAACCCCACA-3′ |

**Supplemental Table 2.** Primer sequences for dual luciferase assays and site-directed mutagenesis

| Gene | Forward/reverse primer sequence | |
|---|---|---|
| *Sglt2* (transformation) | Forward | 5′ -ATATGGTACCACCAAATAAAATCTGAGCATGGA-3′ |
| | Reverse | 5′ -ATATCTCGAGGATTAATGGTTACCTCAGGAGCA-3′ |
| *Sglt2* (sequence) | Forward | 5′ -GGTACCACCAAATAAAATCTGAGCATGGA-3′ |
| | Reverse | 5′ -CTCGAGGATTAATGGTTACCTCAGGAGCA-3′ |
| *Sglt2* (mutagenesis) | Forward | 5′ -AGGATTCAGCTAAATAAAGCTGGAGAA-3′ |
| | Reverse | 5′ -GATCTATCAAGGCCGAAGGCTG-3′ |
| *Glut2* (transformation) | Forward | 5′ -ATATACAGAGCCCACAGAACTAATTTTC-3′ |
| | Reverse | 5′ -ATATGAATTTGCTTAGTAGCCAAAAGGA-3′ |
| *Glut2* (sequence) | Forward | 5′ -CACTAAAATGCTGTGATTCCAACC-3′ |
| | Reverse | 5′ -ATATGAATTTGCTTAGTAGCCAAAAGGA-3′ |

| | | |
|---|---|---|
| *Glut2* (mutagenesis) | Forward | 5′ -TCCTATTCATCCACATTCAGTACAGGA-3′ |
| | Reverse | 5′ - GACCAGCCAGAGTGCTCACTCTA-3′ |

**Supplemental Table 3.** Primer sequences for qPCR

| Gene | Forward/reverse primer sequence | |
|---|---|---|
| *Hprt* | Forward | 5′ -TTGTTGTTGGATATGCCCTTGACTA-3′ |
| | Reverse | 5′ -AGGCAGATGGCCACAGGACTA-3′ |
| *IRS1* | Forward | 5′ -GTTGAGTTGGGCAGAATAGGC-3′ |
| | Reverse | 5′ -GGTATCCACATAGCTTTGACGAG-3′ |
| *IRS2* | Forward | 5′ -CAGTGGGGGCGAACTCTATG-3′ |
| | Reverse | 5′ -CAGGCGTGGTTAGGGAATAAG-3′ |
| *Lrp2* (Megalin) | Forward | 5′ -CAGTGGATTGGGTAGCAGGA-3′ |
| | Reverse | 5′ -GCTTGGGGTCAACAACGATA-3′ |
| *Cubn* (Cubilin) | Forward | 5′ -TCATTGGCCTCAGACATTCC-3′ |
| | Reverse | 5′ -CCCAGACCTTCACAAAGCTG-3′ |
| *Sglt2* | Forward | 5′ -GCAACATCGGCAGCGGTCAT-3′ |
| | Reverse | 5′ -GCGGAGGTACTGAGGCATTGTG-3′ |
| *Glut2* | Forward | 5′ -TCTTCACGGCTGTCTCTGTG-3′ |
| | Reverse | 5′ -AATCATCCCGGTTAGGAACA-3′ |
| *c-Maf* | Forward | 5′ -GGTGGATTGTAGAGGGGAGAG-3′ |
| | Reverse | 5′ -GTTACGGGGGAATTCAGGTT-3′ |
| *Glut1* | Forward | 5′ -ATGGATCCCAGCAGCAAG-3′ |
| | Reverse | 5′ -CCAGTGTTATAGCCGAACTGC-3′ |
| *Sglt1* | Forward | 5′ -CACCATCTTGATCATCTCCTTCCT-3′ |
| | Reverse | 5′ -TGCGTAGACTCCAACACAAAC-3′ |

| | | |
|---|---|---|
| *HNF1α* | Forward | 5′ -AGAGACCTTGGTGGAGTGT-3′ |
| | Reverse | 5′ -GGCAAACCAGTTGTAGACACGC-3′ |
